# IAM‐FIRE: A Climate Emulator–Based Framework to Project Wildfire Impacts and Risks for Integrated Assessment Models

**DOI:** 10.1111/gcb.70951

**Published:** 2026-06-18

**Authors:** Théo Rouhette, Dirk‐Jan Van de Ven, Kanishka Narayan, Claudia Tebaldi, Oliver Perkins, Olivia Haas, Neus Escobar

**Affiliations:** ^1^ Institute of Environmental Science and Technology (ICTA) Universitat Autònoma de Barcelona Cerdanyola del Vallès Spain; ^2^ Basque Centre for Climate Change (BC3) Scientific Campus of the University of the Basque Country Leioa Spain; ^3^ Pacific Northwest National Laboratory (PNNL), Joint Global Change Research Institute (JGCRI) University of Maryland College Park Maryland USA; ^4^ Earth System Science Interdisciplinary Center University of Maryland College Park Maryland USA; ^5^ The Leverhulme Centre for Wildfires, Environment, and Society Imperial College London London UK; ^6^ Department of Geography King's College London London UK; ^7^ School of Environmental Sciences University of East Anglia Norwich UK; ^8^ Geography and Environmental Science University of Reading Reading UK; ^9^ Biodiversity and Natural Resources (BNR) Program International Institute for Applied Systems Analysis (IIASA) Laxenburg Austria

**Keywords:** burned area, climate change, climate emulators, earth system models, fire carbon emissions, integrated assessment models, socioeconomic development, wildfires

## Abstract

Most Integrated Assessment Models (IAMs) underrepresent dynamic feedbacks from climate‐driven disturbances such as wildfires, potentially overestimating the permanence of land‐based carbon sinks. In particular, representing the impacts of forest fires is becoming increasingly important, as these are expected to intensify in the future. We introduce IAM‐FIRE (Integrated Assessment Model—Fire Impacts & Risks Emulator), a novel framework that enables the projection of wildfire burned area (BA) and carbon emissions (CE) directly from IAM outputs. IAM‐FIRE combines a spatial climate emulator, land‐use downscaling, vegetation productivity modelling, and an empirical fire model to generate global annual wildfire impacts for arbitrary socioeconomic and emissions scenarios at 0.5° resolution for the period 2020–2100. Calibrated against GFEDv5 observations and using inputs from the Global Change Analysis Model (GCAM), BA and CE projections are reported for four scenarios: SSP1‐2.6, SSP2‐4.5, SSP3‐6.6 and SSP5‐7.6. The model reproduces historical global trends for both total BA—including the observed global decline since the early 2000s—and forest BA. Projected fire trajectories differ strongly among scenarios: by 2100, total BA range from 441 Mha year^−1^ under SSP1‐2.6 (decline of −2.22 Mha year^−2^ relative to 2020) to 794 Mha year^−1^ under SSP3‐6.6 (increase of +2.3 Mha year^−2^ relative to 2020). Corresponding total CE show a similar divergence by 2100 ranging from 1.8 PgC year^−1^ in SSP1‐2.6 (decline of −9.15 TgC year^−2^ relative to 2020) to 3.6 PgC year^−1^ in SSP5‐7.6 (increase of +12.70 TgC year^−2^ relative to 2020). Socioeconomic development exerts a dominant suppressing effect on wildfire impacts while climate change and CO_2_‐driven increases in vegetation productivity amplify fire risk. Compared to CMIP6 and FireMIP, IAM‐FIRE exhibits greater sensitivity to radiative forcing and a stronger role for human‐driven fire suppression, highlighting structural uncertainties in fire projections. IAM‐FIRE enables systematic exploration of fire–climate–land feedbacks and supports improved assessments of mitigation permanence and climate risks in future integrated scenarios.

## Graphical abstract

Current models can overestimate how much carbon forests can store if they ignore the growing threat of wildfires. To solve this, we develop a new tool called IAM‐FIRE to predict global wildfires and associated carbon emissions through the year 2100. The results show how future fire impacts depend on human choices. If we rapidly cut emissions and ensure human development, global wildfires could actually decrease. However, if emissions keep rising while human development stagnates globally, a surge in forest fires will be observed. Ultimately, IAM‐FIRE can help planners understand fire risks and adjust the use of forest carbon storage accordingly.

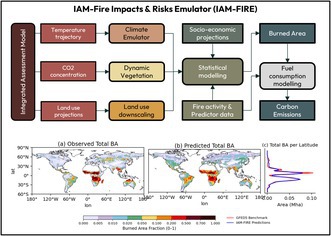

## 1 Introduction

The Intergovernmental Panel on Climate Change (IPCC) often relies on a framework combining Integrated Assessment Models (IAMs), Earth System Models (ESMs), and impact models (IPCC 2023) to explore future mitigation pathways and their implications for climate, ecosystems, and society. IAMs project greenhouse gas (GHG) emissions and removals under different socioeconomic scenarios, providing inputs to ESMs, which in turn generate climate projections for impact models. This linear chain linking IAMs to ESMs and impact models neglects climate feedbacks (processes amplifying or dampening an initial change in radiative forcing) which reduces the internal consistency of projections and has implications for a wide range of biophysical and socioeconomic dynamics (Jones, Adloff, et al. 2024). Accordingly, IPCC AR6 called for improved representation of biophysical climate effects in IAMs to better assess concerns of mitigation permanence (Nabuurs et al. 2022).

Among climate‐driven risks, improving the representation of wildfires is of growing importance given their growing impact on forests' carbon sinks (Anderegg et al. 2020). Related permanence risks are expected to intensify through the 21st century, leading to losses in forest area and carbon stocks (Hermoso et al. 2021). Wildfires pose a worsening threat due to more frequent and severe fire weather, both observed (Bedia et al. 2015; Jones et al. 2022) and projected (Son et al. 2021). Shifts in regional fire regimes can significantly affect the feasibility of large‐scale and long‐term carbon sequestration and the net mitigation potential of forest‐based measures like afforestation/reforestation (Fesenmyer et al. 2025). While an explicit representation of fire dynamics within IAMs is vital to avoid overestimating the potential of forests as a climate mitigation strategy on a warming planet, few established IAMs currently capture the climate feedbacks of worsening fire weather on forest‐based mitigation (Jäger et al. 2024).

Dynamically integrating climate impacts like wildfires into IAMs remains challenging due to technical constraints in the current model chain, particularly the dependency on ESM outputs. While IAM‐generated emissions and land‐use trajectories feed into ESMs to simulate climate and vegetation dynamics, the high computational and labour demands of ESMs restrict simulations to a standardized set of scenarios, most recently following the SSP–RCP framework (Eyring et al. 2016; O'Neill et al. 2017). As a result, only a narrow set of climate responses can currently be passed back into IAMs, making it difficult to ensure a consistent two‐way coupling in which climate change and its impacts meaningfully influence IAM trajectories. Hence, a broader and more flexible range of climate simulations is required to assess how scenario‐dependent climate feedbacks could alter socio‐economic and emission pathways within IAMs.

**FIGURE 1:**
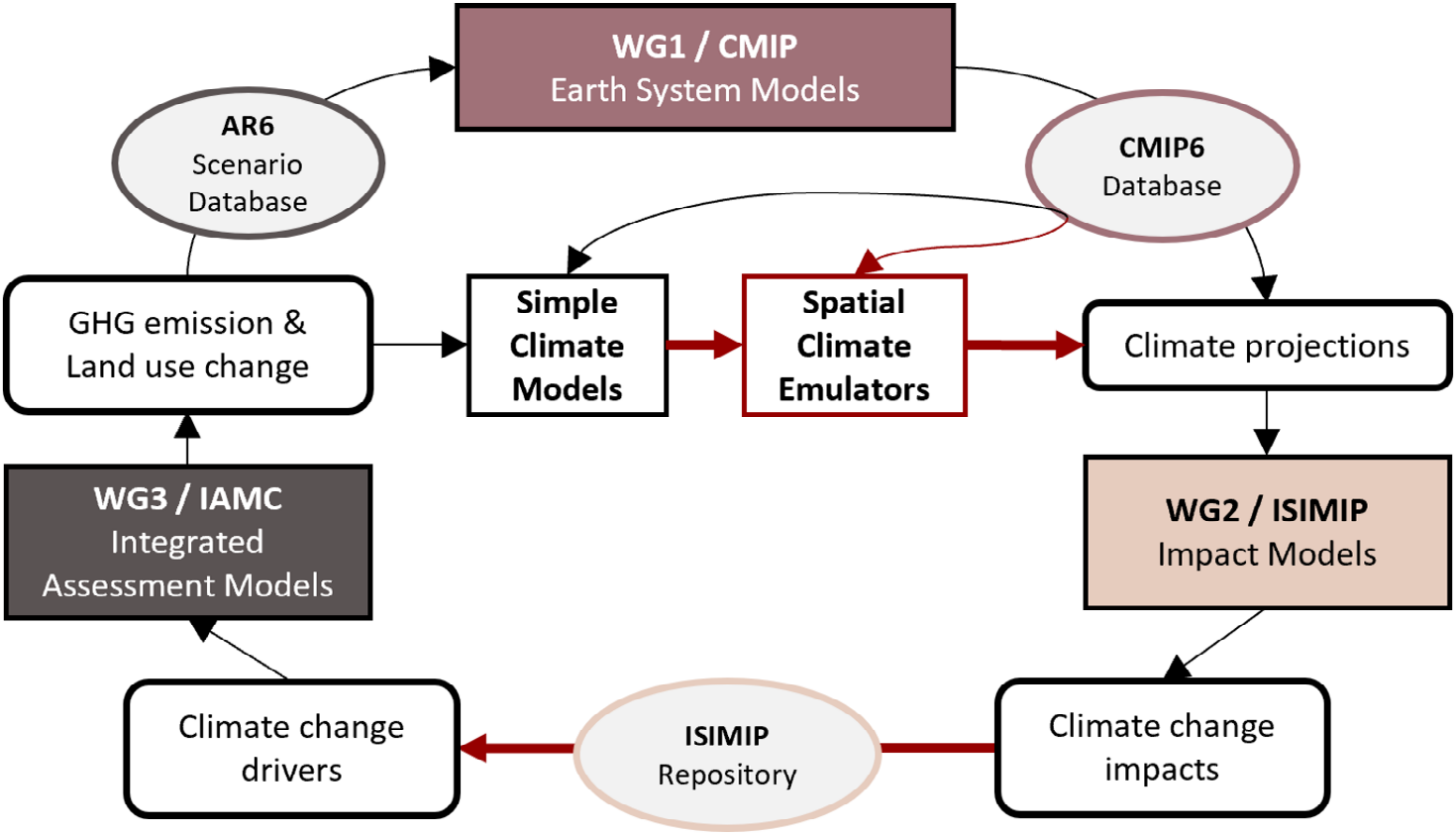
Representation of climate feedback effects in the modelling chain supporting a significant part of the IPCC assessment reports (black lines), where spatial climate emulators help closing the loop (red lines) between Integrated Assessment Models (IAMs, WG3/IAMC) and climate projections from Impact Models (WG2/ISIMIP). SCEs provide spatially resolved climate projections using endogenously derived temperature trajectories from simple climate models and existing climate projections of the Earth System Models (WG1/CMIP) from the CMIP6 database. This facilitates rapid scenario exploration, consistency across working groups, and integration of climate feedbacks.

Spatial climate emulators (SCEs) have been developed to overcome these barriers by providing ESM‐like outputs for arbitrary future scenarios of anthropogenic forcing (Tebaldi et al. 2025). SCEs have become increasingly complex, evolving from simple pattern scaling of temperature and precipitation to reproducing the multidimensional variability and temporal dynamics of full ESM outputs. They derive climate information consistent with the temperature trajectory endogenously produced by simple climate models (SCMs) from IAM outputs (Figure 1). The Fast Assessment for Scenario Trajectories Multi‐emulator Intercomparison Project (FASTMIP) (Seneviratne et al. 2024), a new experimental protocol allowing the fast derivation of climate projections for new emissions scenarios, actively builds on the novel capacities of existing SCEs such as MESMER (Beusch et al. 2020), PRIME (Mathison et al. 2024), or STITCHES (Tebaldi et al. 2022).

Beyond climatic factors, fire regimes are also driven by land use and land use changes (LULUC), topography, vegetation productivity, and human activities (Haas, Keeping, et al. 2024). Most fire‐enabled ESMs estimate burned area (BA) and carbon emissions (CE) using process‐based models which differ in their underlying equations, calibration, and treatment of human drivers. While they reproduce global totals of BA reasonably well, they fail to capture the historic global decline, largely due to underrepresented human suppression (Burton et al. 2024; Li et al. 2024). In parallel, empirical approaches using statistical methods like Generalized Linear Models (GLMs) (Forrest et al. 2024; Haas et al. 2022; Kavhu et al. 2025) have been used in previous studies to predict BA and provide highly interpretable results (Bistinas et al. 2014). However, to our knowledge, they have not yet been combined with climate projections from SCEs.

This study introduces IAM‐FIRE (Integrated Assessment Model—Fire Impacts & Risks Emulator), a novel method designed to predict fire impacts and risks on land systems dynamically through IAM simulations. IAM‐FIRE predicts BA and CE directly from IAM outputs by employing the STITCHES climate emulator to generate the climate variables needed to model fire dynamics. We demonstrate the efficiency and flexibility of this approach to derive consistent outputs using inputs from the Global Change Analysis Model (GCAM) (Calvin et al. 2019). Focusing on fire impacts and risks across multiple SSP–RCP scenarios, we estimate BA and CE at annual resolution on a 0.5° × 0.5° grid (the resolution of ISIMIP3b output variables for fire) for the 2020–2100 period. Additionally, we perform a factorial decomposition to assess the contribution of different drivers and compare results with CMIP6 and FireMIP benchmarks. This methodology provides a computationally rapid and adaptable tool for projecting fire impacts and risks that can facilitate future representation of wildfire feedbacks on land and climate systems within the IAM framework.

## 2 Materials and Methods

### 2.1 Modelling Framework

**FIGURE 2:**
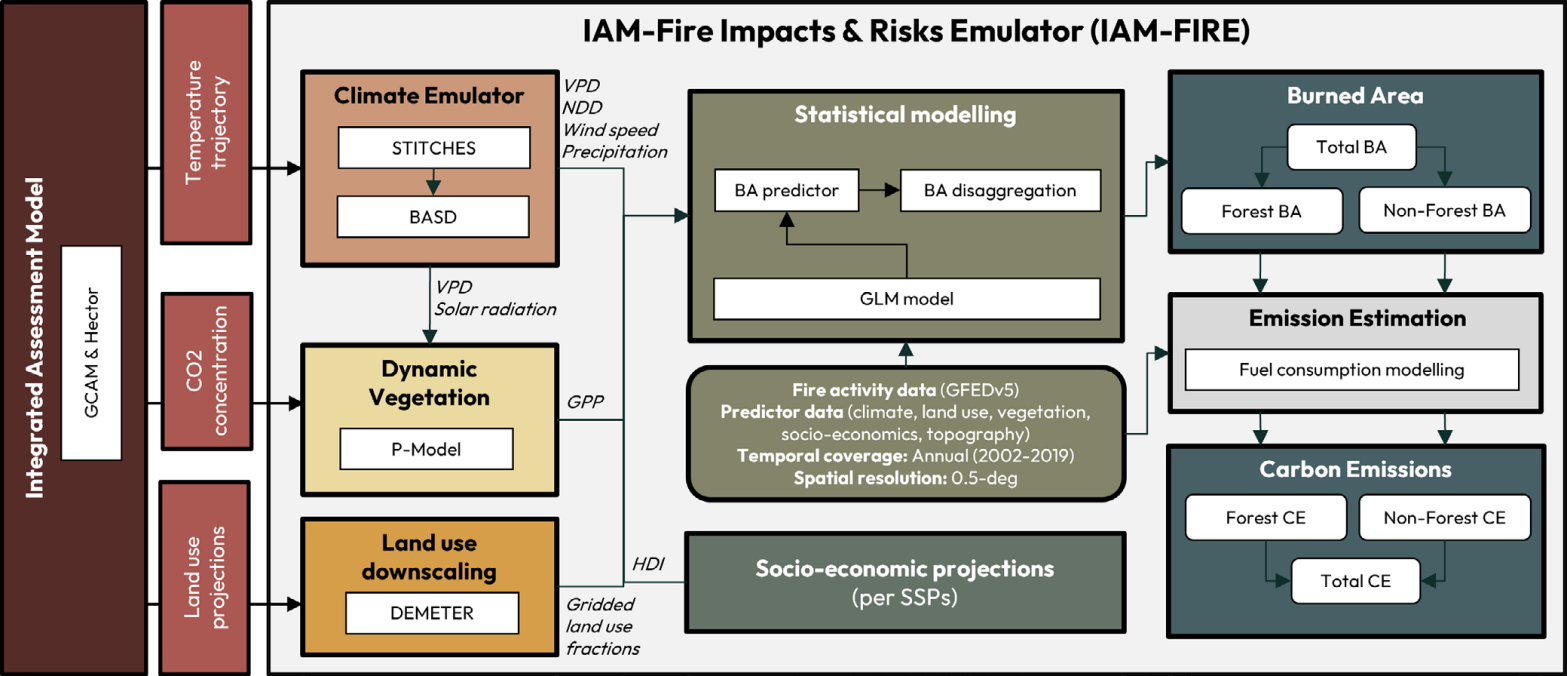
IAM‐FIRE modelling framework to estimate burned area (BA) and carbon emissions (CE) in IAMs combining the Global Change Analysis Model (GCAM) (Section 2.2), the climate emulator STITCHES with Bias‐Adjustment and Statistical Downscaling (BASD) procedure (Section 2.3.1), the vegetation productivity model P‐Model (Section 2.3.2), the land use downscaling tool Demeter (2.3.3), a Generalized Linear Model (GLM) (Section 2.3.4) and a fuel consumption module (Section 2.3.5). GFEDv5, Global Fire Emissions Database version 5.1; GPP, gross primary productivity; HDI, human development index; NDD, number of dry days; SSPs, shared socioeconomic pathways; VPD, vapor pressure deficit.

IAM‐FIRE predicts spatially‐explicit BA and CE for arbitrary IAM scenarios at global scale. The framework combines the climate emulator STITCHES, the land use downscaling tool Demeter, the vegetation productivity model P‐model and a GLM (Figure 2 illustrates the modelling processes of IAM‐FIRE). The framework is calibrated using the fire activity data from the Global Fire Emission Database version 5.1 (hereafter referred to as GFEDv5) (van der Werf et al. 2025). In the following sections, we provide an overview of each methodological step while further details can be found in the Supporting Information.

### 2.2 Land and Temperature: GCAM & Hector

In this study the inputs for IAM‐FIRE are derived from the Global Change Analysis Model (GCAM). GCAM (v8.2) is a dynamic recursive model representing the complex interactions between five major systems—energy, water, land, climate, and the economy (Calvin et al. 2019). GCAM has been widely used for studying climate change mitigation from the land use sector (Rouhette et al. 2024; Zhao, Mignone, et al. 2024), as well as climate impacts on agriculture (Snyder et al. 2020; Zhao et al. 2021), and water systems (Turner et al. 2017). The land and water systems are subdivided into 235 water basins and 32 geopolitical regions that structure the economic and energy systems, resulting in a total of 384 distinct land‐water regions (called land use units, LUTs).

To represent the climate system response to its emissions and land use, GCAM integrates the simple climate model Hector (Dorheim et al. 2024), which projects future GHG concentrations based on a given emissions pathway while simulating the carbon cycle, estimates global mean radiative forcing from these concentrations and short‐lived climate forcers, and translates the radiative forcing into global mean temperature and other Earth system variables (Calvin et al. 2019).

### 2.3 IAM‐FIRE

Three inputs are required to run IAM‐FIRE: the temperature trajectory, the CO_2_ concentration and the land use projections (grassland, shrubland, cropland and forest). In parallel, historic data of fire activity and predictors are compiled over the 2002–2019 period to fit the relationship between observed total BA and the predictors in the statistical model (Table 1 provides the list and sources of the data used in IAM‐FIRE).

**TABLE 1:** Sources of fire response data and predictor variables used for both the historic (2002–2019) and the simulated periods (2020–2100).

Variable	Data source for historic period (2002–2019)	Model used for projection (2020–2100)
*Fire response variable*		
Burned area (BA)	Global Fire Emissions Database (GFED5.1) (van der Werf et al. 2025)	GLM + Post‐processing steps
Carbon emissions (CE)		
*Climate predictors*		
Vapor Pressure Deficit (VPD)	W5E5 v2.0. ISIMIP Repository (Lange et al. 2021)	STITCHES & BASD (Snyder et al. 2024)
Number of dry days (NDD)		
Wind speed		
Precipitation (30‐day rolling sum)		
*Land use predictors*		
Forest	European Space Agency (ESA) Climate Change Initiative (CCI) Plant Functional Types (Harper et al. 2023)	DEMETER (Vernon et al. 2018)
Grassland		
Shrubland		
Cropland		
Grazing pressure	Perkins, Haas, and Millington (2025)	
*Vegetation predictor*		
Gross Primary Productivity (GPP)	P‐Model (Stocker et al. 2020; Wang et al. 2017)	
Temperature	W5E5 v2.0. ISIMIP Repository (Lange et al. 2021)	STITCHES
Vapor Pressure Deficit (VPD)		
Solar Radiation		
Atmospheric CO_2_ concentration	GCAM & HECTOR (Calvin et al. 2019; Dorheim et al. 2020)	
Atmospheric pressure	GMTED2010 Digital Elevation Model (Danielson and Gesch 2011)	
Fraction of absorbed photosynthetically active radiation (fAPAR)	GIMMS FPAR4g (W. Zhao, Zhu, et al. 2024)	
*Socio‐economic predictor*		
Human Development Index (HDI)	Kummu et al. (2018)	Perkins et al. (2024)
*Topographic predictors*		
Topographic Position Index (TPI)	Amatulli et al. (2018)	
Vector ruggedness measure (VRM)		
*Fuel consumption*		
Fuel load (FL)	ECMWF Fuel Characteristics V1 (McNorton and Di Giuseppe 2024)	
Combustion completeness (CC)	Ranges per biomes and fuel pools (van Wees et al. 2022)	

*Note:* Detailed processing steps are provided in the Supporting Information.

#### 2.3.1 Climate Emulation: STITCHES & BASD

The global average temperature projections of Hector are fed into the climate emulator STITCHES (Snyder et al. 2024). STITCHES utilizes existing archives of Earth system models' (ESMs) scenario experiments to generate ESM‐like outputs for new scenarios or to enhance existing initial‐condition ensembles. It preserves the key characteristics of the ESM output it aims to replicate (multivariate, spatially detailed, and high‐frequency) while capturing both the forced response and the surrounding internal variability (Tebaldi et al. 2022). STITCHES follows a time‐slicing approach which takes several time windows of gridded, multivariate outputs from ESMs and recombines them into new instances of transient trajectories for future periods by stitching them together on the basis of the global surface air temperature (GSAT) derived from the GCAM scenario.

We selected two ESMs that provided enough experiments in the Pangeo‐hosted CMIP6 database at the daily scale required for the analysis: CanESM5 (Swart et al. 2019) and MPI‐ESM1‐2‐LR (Gutjahr et al. 2019) (Table S1). We use STITCHES to derive five climatic variables provided by CMIP6 models in the period 2015–2100: temperature (tas), relative humidity (hurs), precipitation (pr), wind speed (sfcWind), and solar radiation (rsds). Climate simulation data provided by CMIP6 are characterized by systematic biases relative to the climate observation data. In order to remove these biases, a bias adjustment is required which involves two distinct procedures: (i) the actual bias adjustment at the spatial resolution of the simulation data and (ii) a statistical downscaling to the spatial resolution of the observation data. We perform both bias adjustment and statistical downscaling (BASD) following the approach described by Lange (2019). The observational data used for the BASD procedure is the W5E5 v2.0 dataset compiled to support the bias adjustment of climate input data for Phase 3b of the Inter‐Sectoral Impact Model Intercomparison Project (ISIMIP) (Frieler et al. 2024). The same dataset is used for the statistical model (described below) of IAM‐FIRE to ensure consistency between historical observations and future projections of climate drivers.

For each scenario and ESM the emulated and bias‐adjusted climate variables provided by STITCHES are used to derive the final climatic drivers: monthly temperature and relative humidity are used to compute the vapor pressure deficit (VPD), daily precipitation is used to compute the monthly number of dry days (NDD) and the 30‐day precipitation rolling sum and monthly temperature and wind speed are used to compute the mean wind speed of the hottest month. Solar radiation is used to model gross primary productivity (GPP), as described below.

#### 2.3.2 Vegetation Projection: P‐Model

Mean annual GPP is used as an index of vegetation productivity (Krause et al. 2022) and is derived from the P‐Model (Stocker et al. 2020; Wang et al. 2017). Using the inputs described in Table 1, it estimates GPP as the product of light use efficiency (LUE) of photosynthesis and absorbed radiation. These variables represent the key environmental controls on photosynthesis: light availability determines the energy input for carbon fixation, while temperature, VPD, and CO_2_ concentration regulate stomatal behavior and biochemical efficiency. Together, they capture the eco‐physiological constraints that govern LUE and therefore GPP.

The P‐Model is used for both the historic and simulated period to ensure consistency between both periods. CO_2_ concentrations are extracted from Hector while the VPD and solar radiation are emulated with STITCHES. Solar radiation is converted to photosynthetic photon flux density using established constants (Haas et al. 2022) and elevation to atmospheric pressure using a dedicated function of the P‐Model. We assumed a constant fAPAR by using monthly climatological means from the historical period, meaning that potential future increases in canopy density or vegetation greenness are not represented (Gao et al. 2023). As a result, our projections reflect changes driven solely by environmental controls on LUE and may underestimate future GPP in regions where fAPAR is expected to increase (see a sensitivity analysis on the impact of this assumption on historic GPP and BA predictions in the Supporting Information).

To account for periods of high versus low GPP, the seasonality index is estimated for each year and grid cell as the relative intra‐annual amplitude of the mean GPP in each calendar month (see Equation 1 in Supporting Information). The seasonality index of GPP is used as an additional predictor of the statistical modelling.

#### 2.3.3 Land Use Downscaling: Demeter

To represent the role of land use in fire regimes, the aggregated land use projections of GCAM are downscaled at the grid level (0.5‐deg resolution) with Demeter (Vernon et al. 2018). Demeter is an open‐source spatial disaggregation model providing gridded land cover change (LULUC) products derived directly from IAMs in a variety of formats and resolutions commonly used by ESMs. Demeter has been used to generate a new global gridded land use dataset at a high spatial resolution (Chen et al. 2020). Demeter utilizes a base land cover map at a target resolution as a reference and strategically distributes projected land type area changes from models (e.g., GCAM) to target grid cells according to a set of user‐defined rules and spatial constraints (Chen et al. 2019).

In this study, we update the base map of Demeter with the ESA CCI Land Cover data (Figure S1, Tables S2 and S3). Since CGAM land module is calibrated on HYDE 3.2 (Klein Goldewijk et al. 2017), we employ an established methodology to reproject GCAM land use outputs onto the novel ESA base map (Chen et al. 2020). The downscaled results are grouped into broad land use categories: forest, grassland, shrubland, cropland, and other land (tundra, rock, ice, desert, and urban) at the 0.5‐degree resolution and linearly interpolated from a 5‐year time step to annual time step for the period 2015–2100.

#### 2.3.4 Statistical Modelling of Burned Area: GLM

To predict future BA, a statistical model is trained on historical data over 2002–2019. Following previous studies (Haas et al. 2022; Kavhu et al. 2025), we use a generalized linear model (GLM) to estimate BA fractions from a range of predictors. BA and CE are derived from GFED5 which provides data at 0.25° × 0.25° resolution for the period from 1997 to 2022 (van der Werf et al. 2025). Since IAM‐FIRE focuses on wildfires, we do not account for human use of fire related to agricultural burning or deforestation fires.

For the drivers of burned area, predictor variables are grouped in five categories: climatic, land use, vegetation, topographic, and socio‐economic. To represent climatic drivers, we use VPD, NDD, the 30‐day precipitation rolling sum, and wind speed. For the simulated period (2020–2100), these drivers are estimated from the bias‐adjusted and statistically downscaled projections from STITCHES for the selected ESMs. For drivers related to vegetation and land uses, we use GPP, the GPP seasonality index, grazing pressure (average computed over the 2002–2014 period) from Perkins, Haas, and Millington (2025) and fractions of grassland, shrubland, forestland, and cropland from the ESA CCI product for Plant Functional Types aggregated at 0.5‐degree resolution by Harper et al. (2023). For topography, we use topographic positioning index (TPI) and vector ruggedness measure (VRM), which are static during the simulated period. For human influence, Human Development Index (HDI) is selected as it captures the country's social and economic development levels. Subnational HDI projections were obtained per SSPs from Perkins et al. (2024).

To reduce skewedness, we log‐transform VPD, NDD, wind speed, precipitation rolling sum, and GPP. We run the GLM using a quasi‐binomial function with a logit‐link function adapted to the nature of the response variable (BA fraction) ranging from 0 to 1. Since some fire predictors are known for having non‐linear relationships to BA, we use a Generative Additive Model (GAM) to identify non‐linear relationships and we add polynomial terms to the corresponding predictors in the GLM. We also test additional predictors like population density and a land fragmentation proxy (Table S9). We identify the final formula by running different models which are evaluated and compared using the coefficient of determination (R^2^), the normalized mean error (NME), and root mean square error (RMSE). We finally run a hindcast experiment using 3‐year rolling windows to ensure the out‐of‐sample validity of the prediction. Details and equations are provided in Supporting Information.

In GLMs, the relative importance of each predictor is assessed using absolute t‐values (the fitted regression coefficient of each predictor divided by its standard error) which are reported along with summary metrics for the best model. We perform multicollinearity analysis looking at variance inflation factors (VIF) to assess redundancy between predictor variables.

Forest BA is derived from total BA based on the average proportion of forest BA per grid cell from GFEDv5 historical data. It is assumed that this proportion remains constant over the simulated period (Figure S2).

#### 2.3.5 Modelling of Carbon Emissions

CE from biomass combustion are calculated by projecting fuel consumption (FC) which is then multiplied by BA (Supporting Information Section 1.2.6). FC depends on the biomass available to burn (fuel load, FL) and the fraction of the biomass that is combusted (combustion completeness, CC). FL data is obtained from the global fuel characteristic dataset (McNorton and Di Giuseppe 2024) and CC values from van Wees et al. (2022) (Table S4). CC was then scaled linearly by VPD values to account for the impact of dryness/wetness of the fuel. By combining FL data with the scaled CC factors, we derive spatially explicit and climate responsive FC values for the four fuel types (leaf, stem, litter and coarse wood debris) over the simulated period (Figure S3). Forest FC is obtained by summing all four fuel types and non‐forest FC by summing litter and leaf.

To ensure consistency between our values and GFEDv5 FC values (Figures S3 and S4), which are constrained by field measurements and broadly accepted references for CE, we estimate a change ratio for our projected FC and multiply the GFEDv5 FC values in 2019 by the ratio per year for both land use categories in IAM‐FIRE (forests and non‐forests) (Zhao et al. 2025). This implies that FC projections are aligned with the GFEDv5 historical benchmark used in the GLM and maintain dynamic changes under diverging future climate conditions. Trends of global FC are presented in the results with weighted averages using the fixed BA data for 2019 in order to isolate the FC signal across scenarios with different future BA trajectories.

### 2.4 Scenario Design

The estimates of BA and CE are computed for four scenarios ranging from low to high emissions. We run SSP1‐2.6 and SSP2‐4.5 reference scenarios as well as reference scenarios for SSP3 and SSP5, which in GCAM v8.2 only reach radiative forcings of 6.6 and 7.6 by 2100, respectively (Figure S5). Since STITCHES emulates specific ESMs, we model each scenario using the 2 ESMs presented above, addressing structural uncertainties in the modelling of the climate system. In the absence of a meaningful distribution with 2 ESMs, our main results report the multi‐model mean (MME) along with the range across the models.

### 2.5 Factorial Decomposition

To assess the relative importance of predictors on the total BA per scenarios, we run factorial simulations for both historic and future periods following the approach of Wu et al. (2021) which differentiates limiting factors and drivers (Table S5). We grouped the predictors into climatic (VPD, NDD, wind speed, precipitation), land use (grassland, shrubland, cropland, grazing pressure), vegetation (GPP) and socio‐economic (HDI) groups. For each group, we test its role as a driver of BA by allowing the variables of the group to vary while fixing the variables of all the other groups at their base year values; and as a limiting factor of BA by fixing its value at the base year while allowing the other groups to vary. The base year is 2002 for the historic period and 2020 for the future period.

### 2.6 Comparison With CMIP6 and FireMIP

We compare the total BA results with BA projections from CMIP6 and FireMIP runs. For CMIP6, we select the total BA outputs from four ESMs listed in Li et al. (2024): CESM2, CESM2‐WACCM, CMCC‐ESM2, Nor‐ESM2‐LM. For FireMIP, we select the total BA for three impact models: CLASSIC, ECM‐ELA and VISIT. For each model we extract BA for the following experiments when available: historical, SSP1‐2.6, SSP2‐4.5, SSP3‐7.0 and SSP5‐8.5 (although GCAM 8.2 produces baseline scenarios for SSP3 and SSP5 that reach intermediate levels of radiative forcing, as SSP3‐6.6 and SSP5‐7.6). We compare the IAM‐FIRE BAs with the benchmark BAs looking at global totals and at temporal linear trends computed by ordinary least square OLS (and tested for significance using the Mann‐Kendall test).

## 3 Results

### 3.1 Historical Burned Area and Carbon Emissions

**FIGURE 3:**
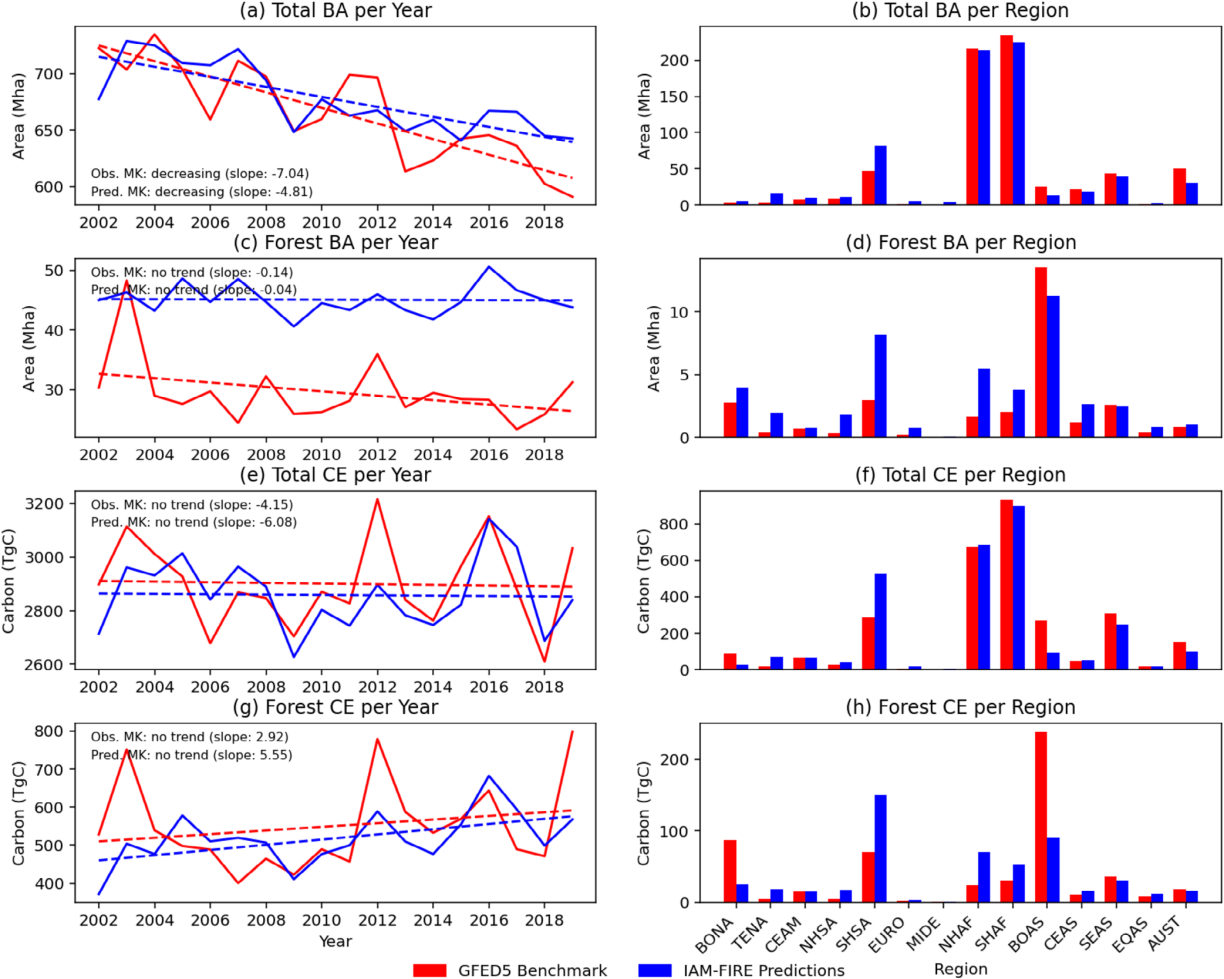
Temporal trend for total BA time series (a) and regional average (b), forest BA time series (c) and regional average (d), total CE time series (e) and regional average (f), and forest CE time series (g) and regional average (h). Regional averages are computed over 2002–2019 for burned areas and carbon emissions. AUST, Australia and New Zealand; BOAS, Boreal Asia; BONA, Boreal North America; CEAM, Central America; CEAS, Central Asia; EQAS, Equatorial Asia; EURO, Europe; MIDE, Middle East; NHAF, Northern Hemisphere Africa; NHSA, Northern Hemisphere South America; SEAS, Southeast Asia; SHAF, Southern Hemisphere Africa; SHSA, Southern Hemisphere South America; TENA, Temperate North America. MK refers to the Mann‐Kendall test.

**FIGURE 4:**
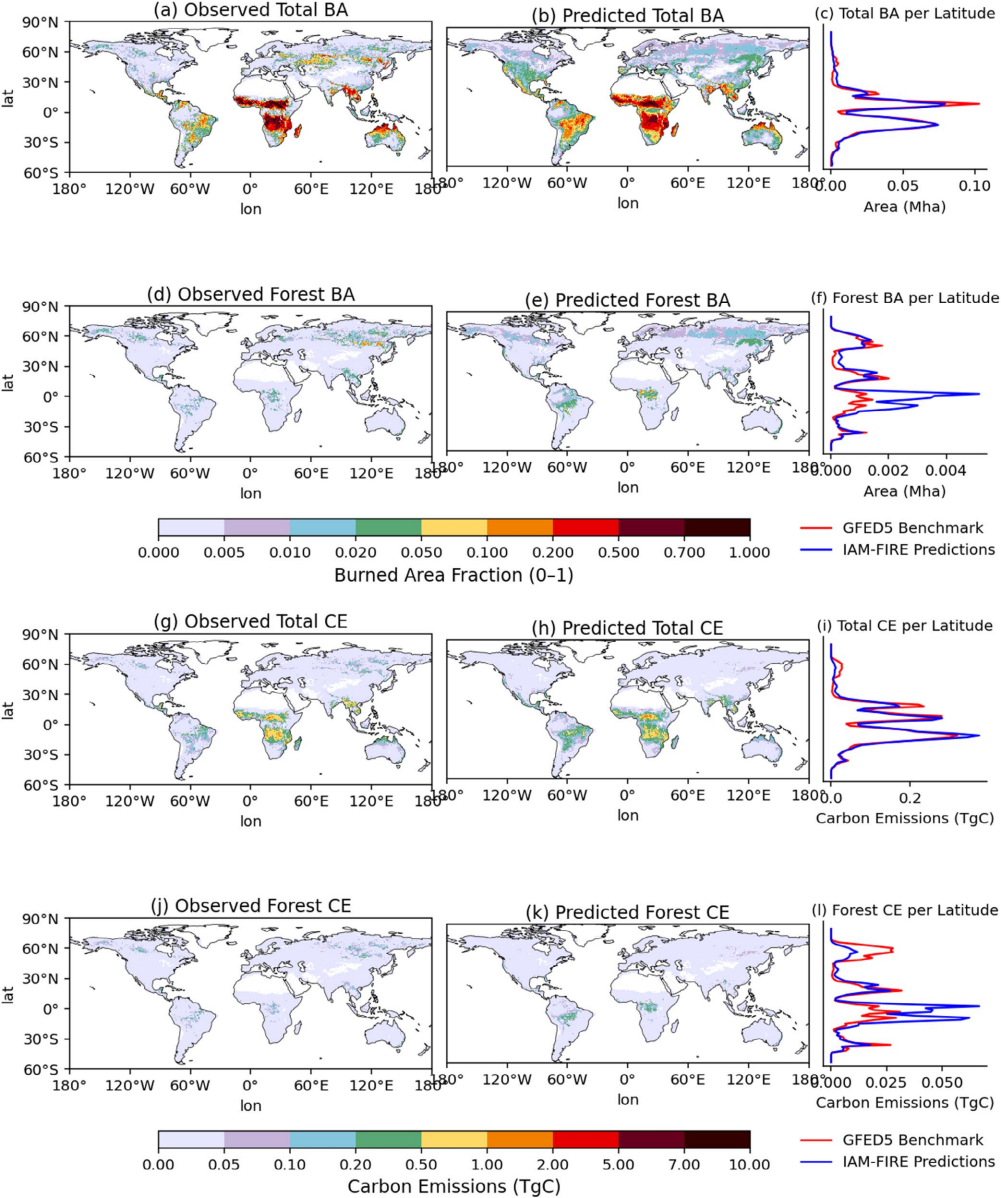
Average annual values of observed and predicted response variables and mean latitudinal distributions over historic period (2002–2019) for total BA (a–c), forest BA (d–f), total CE (g–i), and forest CE (j–l). Maps for BA are shown in fraction (0–1) and latitudinal bands in Mha. Carbon emissions are reported in TgC.

BA and CE predictions for historical periods are shown in Figure 3. Predicted total BA adequately captures the decline reported by GFED5 in the 2002–2019 period (Figure 3a). The spatial distribution is well captured by the GLM with minor differences in latitudinal bands (Figure 4a–c). Aggregated per GFED regions, higher estimates are observed in SHSA (South Hemisphere South America) (47 Mha year^−1^ observed vs. 81 Mha year^−1^ predicted) (Figure 3b). For total CEs, both the predictions and observations of total CE show an absence of trend in historic period, and IAM‐FIRE captures well global totals (2.86 PgC year^−1^ predicted vs. 2.9 PgC year^−1^ observed) (Figure 3e,f).

For forests, predicted BA captures the absence of trend in the 2002–2019 period (Figure 3c). On average, global forest BA is higher by 15 Mha (30 in GFED vs. 45 in IAM‐FIRE), due to the higher values in SHSA and to a lesser extent in NHAF (North Hemisphere Africa) and SHAF (South Hemisphere Africa) (Figure 3d). For forests CE, both predictions and observations report no statistically significant trend. Despite the agreement on global totals, IAM‐FIRE has higher forest CE in the SHSA region (0.07 TgC year^−1^ observed vs. 0.15 TgC year^−1^ predicted) and lower in boreal regions (0.32 TgC year^−1^ observed vs. 0.12 TgC year^−1^ predicted across BONA and BOAS) (Figure 3g,h).

The regional discrepancies highlighted can be explained by several factors (Figures S6–S9). The results in SHSA could be caused by an underrepresentation of suppressing effects like moisture constraints and land fragmentation which strongly limit BA in tropical forests. Due to the multiplicative nature of the CE calculations, moderate gaps in total BA can result in large differences in CE estimates through error propagation. On the contrary, in boreal forests the model aligns with observed BA, but a spatial misallocation of BA towards lower‐biomass areas explains why CE remains underestimated, especially in the case of forests CE. In addition, contemporary fires in boreal forests are dominated by very large and intense fire years (such as 2003 and 2016—see Supporting Information). In common with many fire models, IAM‐FIRE cannot fully capture these dynamics (Hantson et al. 2024).

**FIGURE 5:**
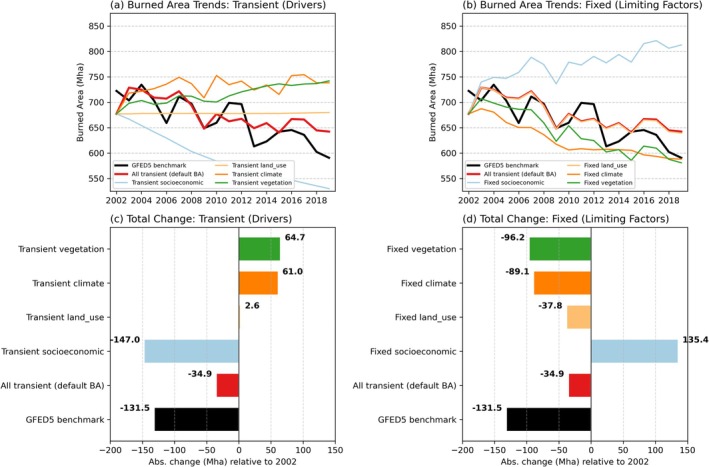
Factorial decomposition of total burned area over the historic period (2002–2019). Temporal trends for analysis of drivers (a) and of limiting factors (b), and absolute change in 2019 relative to 2002 for drivers' mode (c) and limiting factors' mode (d). Note that in 2002 (base year), the observed burned area (BA) in GFED5 benchmark is 722 Mha while the IAM‐FIRE predicted BA is 677 Mha.

Overall, the GLM performs well with high predictive power: a pseudo *R* ^2^ of 0.75, NME of 0.43 and RMSE of 0.06 (see Table S7 for summary statistics and Figure S10 for partial residual plots). The rolling window hindcast results demonstrate that the fire prediction model is highly robust to out‐of‐sample tests throughout the historic period (see Supporting Information Section 2.1.3). The factorial decomposition illustrates the relative importance of each variable group in the observed global decline of BA. Without improvements in HDI (Figure 5b,d), global BA would have increased by 135.4 Mha between 2002 and 2019, demonstrating the major role of socio‐economic development on observed trends. Both climate and vegetation variables have enhanced global BA in comparable magnitudes: all else equal, dynamic climate would have increased BA by 61 Mha and dynamic vegetation by 64.7 Mha between 2002 and 2019 (Figure 5a,c).

### 3.2 Future Burned Area and Carbon Emissions

We report future global BA and CE trends for total fires and forest fires as ensemble means across the 2 ESMs used to emulate climate variables (CanESM and MPI) for the four scenarios over the 2020–2100 period.

**FIGURE 6:**
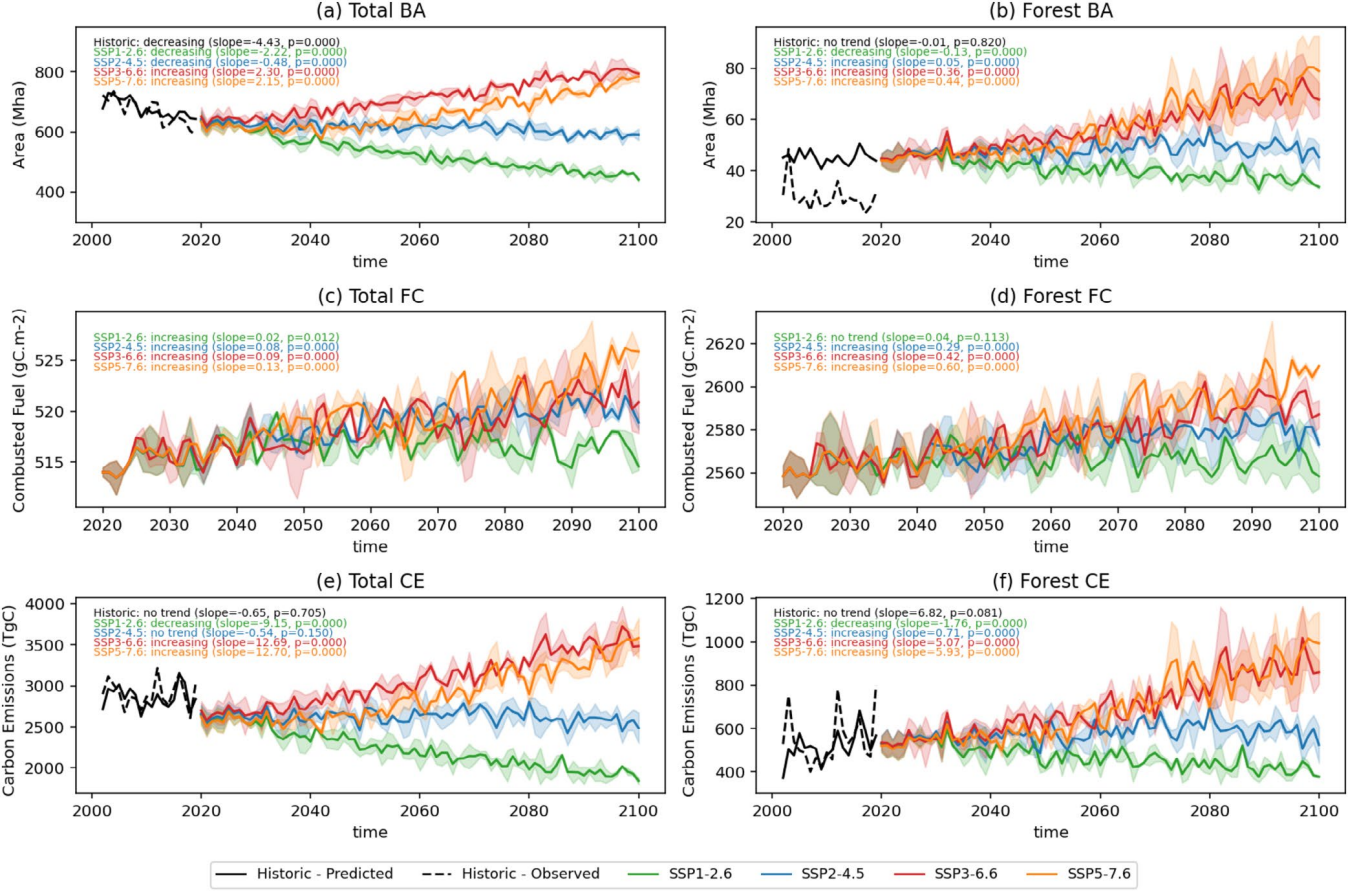
Observed and projected (2002–2100) burned area (top row) and carbon emissions (bottom row) and projected (2020–2100) fuel consumption (middle row) for total land (left column) and forests (right column). The shaded areas represent the range around the mean of the 2 ESMs (CanESM and MPI).

**FIGURE 7:**
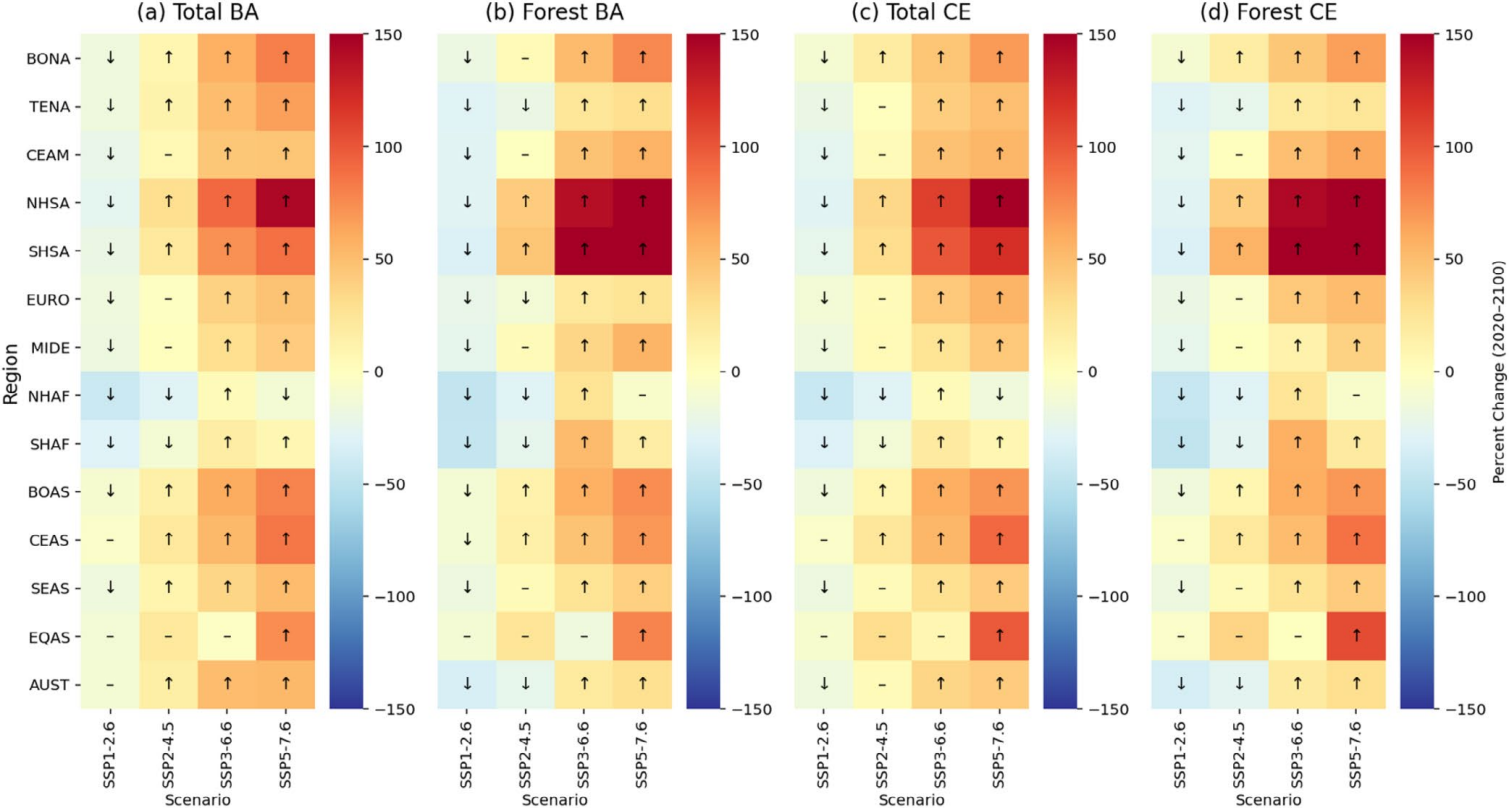
Heatmap of trends of total BA, forest BA, total CE and forest CE per region and scenarios in percent change in 2100 relative to 2020. Arrows represent the results of the Mann‐Kendall test (statistically increasing, decreasing or no trends). AUST, Australia and New Zealand; BOAS, Boreal Asia; BONA, Boreal North America; CEAM, Central America; CEAS, Central Asia; EQAS, Equatorial Asia; EURO, Europe; MIDE, Middle East; NHAF, Northern Hemisphere Africa; NHSA, Northern Hemisphere South America; SEAS, Southeast Asia; SHAF, Southern Hemisphere Africa; SHSA, Southern Hemisphere South America; TENA, Temperate North America.

The scenarios show strongly diverging trends of total BA at the global level (Figure 6a,b). SSP1‐2.6 shows a sharp decline in BA, reaching 440 Mha year^−1^ (average trend of −2.22 Mha year^−2^) for total BA and 34 Mha year^−1^ (average trend −0.13 Mha year^−2^) for forest BA by 2100, due to high human development and strong mitigation of GHG emissions. Africa (both NHAF and SHAF) contributes most to the decline in both total and forest BA, while most regions (11 out of 14) exhibit declining trends (Figure 7a,b). SSP2‐4.5 has a decreasing trend of −0.48 Mha year^−2^ for total BA, reaching 572 Mha year^−1^ by 2100, but an increasing trend of 0.05 Mha year^−2^ for forest BA reaching 50 Mha year^−1^ by 2100 due to lower HDI improvements (Figure S17f) and higher GHG emissions. Multiple regions will switch to increasing trends compared to SSP1‐2.6 and only African regions will still have decreasing total BA (Figure 7a,b). This scenario is marked by strong divergences in regional trends due to both moderate human development and climate impacts. SSP5‐7.6 closely follows the trend observed in SSP2‐4.5 up until mid‐century due to a similar HDI increase, but diverges around 2060 as climate impacts significantly worsen. Total BA increases much faster (2.15 Mha year^−2^) than in SSP2‐4.5—reaching 765 Mha year^−1^ by 2100—and forest BA globally increases up to 65 Mha year^−1^ at a rate of 0.44 Mha year^−2^. This scenario is marked by increases in total and forest BA in all regions except NHAF. SSP3‐6.6 shows the strongest positive trend for total BA with 2.30 Mha year^−2^ reaching 788 Mha year^−1^ by 2100 due to the combined effect of high climate change impacts and the lowest HDI increases among the four scenarios due to underlying regional rivalry assumptions. However, forest BA increases less than in SSP5‐7.6, reaching 61 Mha year^−1^ by 2100 with a trend of 0.36 Mha year^−2^. All regions but EQAS will see increases in both total and forest BA. Across the high‐emission scenarios, South American regions exhibit the highest increases in fire impacts in percent change relative to 2020 (Figure 7a,b).

IAM‐FIRE projects changes in FC (biomass burned annually per m^2^ of BA) depending on the meteorological conditions as CC increases along with VPD—the indicator used as a proxy of fuel dryness in this study. FC is projected to increase in all scenarios, albeit with different magnitudes depending on the climate trajectory (Figure 6c,d). For total fires, the projected FC increases range from 0.02 gC m^2^ year^−2^ for SSP1‐2.6 to 0.13 gC m^2^ year^−2^ for SSP5‐7.6. For forest fires, IAM‐FIRE reports an increasing trend in FC ranging from 0.04 gC m^2^ year^−2^ for SSP1‐2.6 to 0.60 gC m^2^ year^−2^ for SSP5‐7.6. Unlike BA results, SSP5‐7.6 has higher increases in FC than SSP3‐6.6 since FC is only impacted by VPD and is independent from fire suppression processes such as HDI or LULUC.

The CE projections arise from both the BA and the FC trends (Figure 6e,f). The low emission scenario has statistically significant declines in CE from fires globally. SSP1‐2.6 shows the sharpest decline, reaching 1.8 PgC year^−1^ by 2100 with a trend of −9.15 TgC year^−2^ for total CE and 372 TgC year^−1^ with a trend of −1.76 TgC year^−2^ for forest CE. The medium emission scenario SSP2‐4.5 has no significant trend for total CE but forest CE will increase by 0.71 TgC year^−2^ up to 442 TgC year^−1^ by 2100 (Figure 7c,d). High emission scenarios have increasing CE for both total and forest wildfires. Both SSP5‐7.6 and SSP3‐6.6 show a trend of 12.7 TgC year^−2^ for total CE, reaching 3.6 and 3.5 PgC year^−1^ by 2100 respectively. Like for BA projections and combined with a higher FC due to fuel dryness, forest CE is higher in SSP5‐7.6 than SSP3‐6.6, with 849 TgC year^−1^ (trend of 5.93 TgC year^−2^) and 806 TgC year^−1^ (trend of 5.07 TgC year^−2^) by 2100, respectively. Total CE is expected to significantly increase in most regions in these scenarios, in sharp contrast with SSP1‐2.6 and SSP2‐4.5 (Figure 7c,d).

### 3.3 Contributions of the Drivers of Burned Area

**FIGURE 8:**
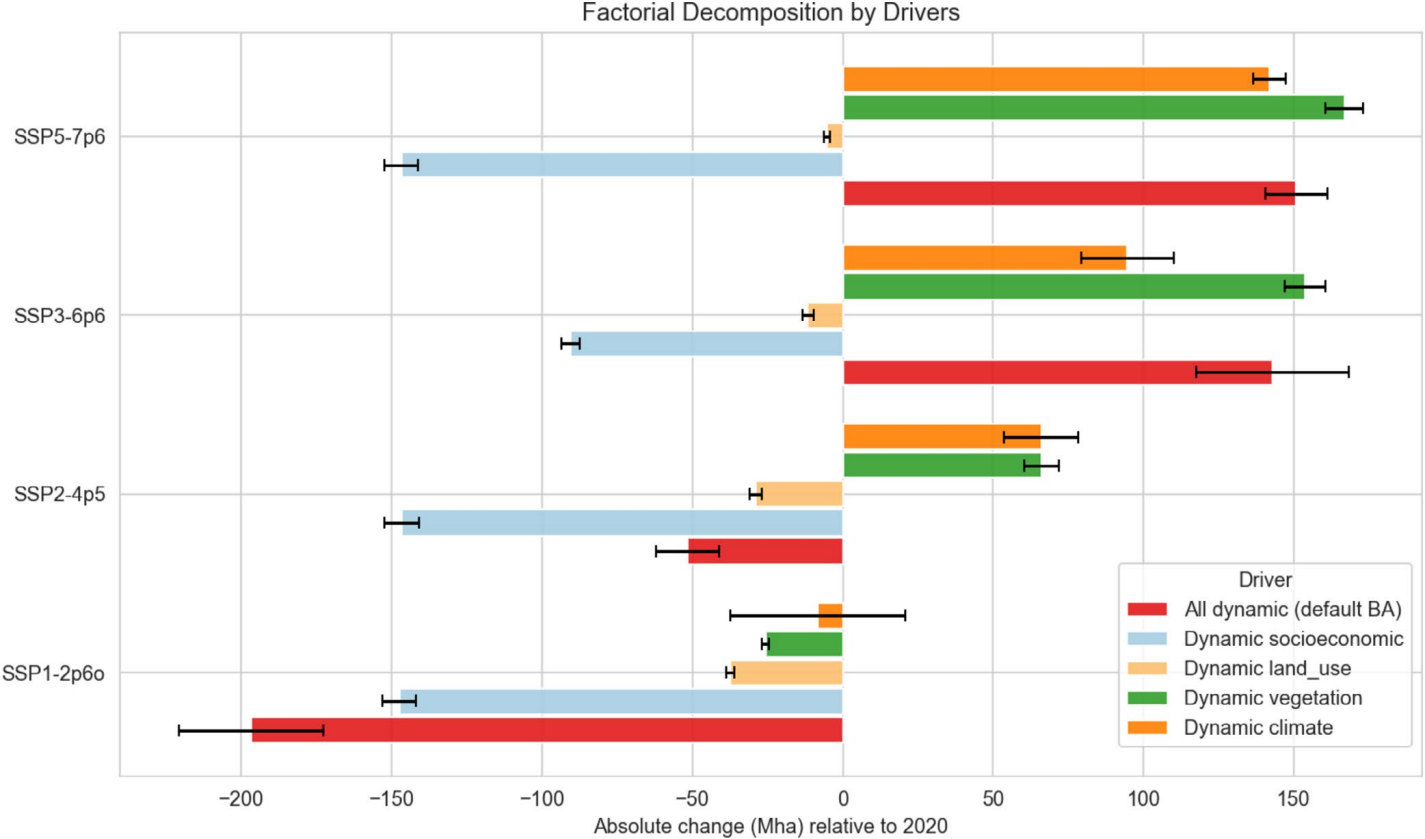
Factorial decomposition of drivers of total BA across scenarios. The absolute change of total BA (Mha) relative to 2020 is shown per scenario for default BA and four decomposition runs where only the group of drivers is allowed to vary, keeping others fixed at the 2020 value: dynamic socioeconomic (HDI), dynamic land use (cropland, grassland), dynamic vegetation (GPP), and dynamic climate (VPD, NDD, NDD seasonality, and precipitation). Error bars show the range (maximum and minimum) from the two ESMs (CanESM and MPI).

We performed a factorial decomposition analysis of the drivers of total BA projections (Figure 8) to better understand how changes in climate, vegetation, land use, and socio‐economic factors influence the projected fire impacts. Details on time series of predictors (Figure S17) and on the factorial decomposition (Figures S18–S20) are provided in Supporting Information Section 2.5.

#### 3.3.1 Socio‐Economic Development

HDI is the main driver of total BA results at low and medium emission levels, which reflects the high t‐value of the GLM for the historic period and the significant range of projections from the SSP assumptions. HDI has a non‐linear relationship with BA where its BA‐reducing effects are dampened at high development levels, demonstrating the limits to fire suppressing strategies. Such non‐linearity coupled to large differences in projected patterns across SSPs lead to significant divergence in the relative strength of fire suppression processes in IAM‐FIRE, both globally and regionally. To illustrate this, fixing all drivers but HDI leads to significant reduction of global total BA compared to baseline values at 2020 under all scenarios. SSP2‐4.5, SSP1‐2.6, and SSP5‐7.6 have similar global outcomes (−146 Mha year^−1^ relative to 2020), although SSP2‐4.5 has lower HDI projections. This result captures how, past a threshold, further HDI improvement does not translate into BA reduction. Still, SSP3‐6.6, which projects the lowest increases in HDI across SSPs, has lower suppressing effects with −90 Mha year^−1^ relative to 2020. Across the scenarios, the regions driving these reductions are African regions and to a lesser extent Southeast Asia and the Middle East. On the contrary, a globally stagnant HDI coupled with high emission (SSP5‐7.6) would lead to significantly worsening fire impacts with total BA increasing by +335 Mha year^−1^ (Figure S19) relative to 2020.

#### 3.3.2 Land Use and Land Use Changes

LULUC, which refers to the way specific areas of land are used or managed by humans and their alterations through time, has suppressing effects on global fire projections across the scenarios. SSP1‐2.6 and SSP2‐4.5 are the scenarios with the largest suppressing impacts of LULUC due to large decreases in grassland and shrubland areas (Figure S15j). The effects of LULUC are moderate in high‐emission scenarios due to a relatively lower decrease in grassland (SSP5‐7.6) or a larger increase in cropland (SSP3‐6.6). Across scenarios, LULUC has the largest effects relative to other drivers in Europe and Temperate North America. Globally, high deforestation is observed for SSP3‐6.6, while SSP1‐2.6 yields significant afforestation/reforestation, with both SSP2‐4.5 and SSP5‐7.6 maintaining global forest areas around 2020 values (Figure S17).

#### 3.3.3 Climatic Factors

Climatic factors capture the combined effects of VPD, NDD, and precipitation on future BAs. Globally, the average maximum VPD will increase proportionally to the RCP levels. VPD will only decrease in SSP1‐2.6, going back to historical levels (2020 baseline) in the second half of the century. While climatic impact on BA is small and highly uncertain for SSP1‐2.6 (−8 Mha year^−1^), it becomes a positive driver of total BA reaching a maximum of 142 Mha year^−1^ by 2100 in SSP5‐7.6. Climatic factors are the main drivers in several northern regions (BONA, TENA, Europe) and tropical regions (NHSA, Southeast Asia) at high emissions levels (Figure S18), although the effect is associated with relatively high uncertainty across the two ESMs.

#### 3.3.4 Vegetation Productivity

Like climatic factors, vegetation productivity is driven by changes in CO_2_ concentration and has a positive impact on total BA. GPP growth is enhanced by CO_2_ fertilization and higher RCP levels lead to increases in weighted average GPP values across ecosystems. SSP1‐2.6 is the only scenario where vegetation productivity has a moderately suppressing impact on total BA. CO_2_ fertilisation is a stronger driver of total BA than climate change in high levels of warming (RCP 6.6 and 7.6). Regional results show a variety of distinct relative contributions between climatic factors and vegetation productivity at all warming levels, illustrating the complex relationships between fuel load and fuel dryness dynamics as driving factors of BA.

### 3.4 Comparison with CMIP6 and FireMIP

To shed light on the differences between IAM‐FIRE and process‐based fire models, we compare existing projections from CMIP6 and FireMIP with the results of IAM‐FIRE for the four scenarios closest to the ones we ran in GCAM (SSP1‐2.6, SSP2‐4.5, SSP3‐7.0, SSP5‐8.5).

**FIGURE 9:**
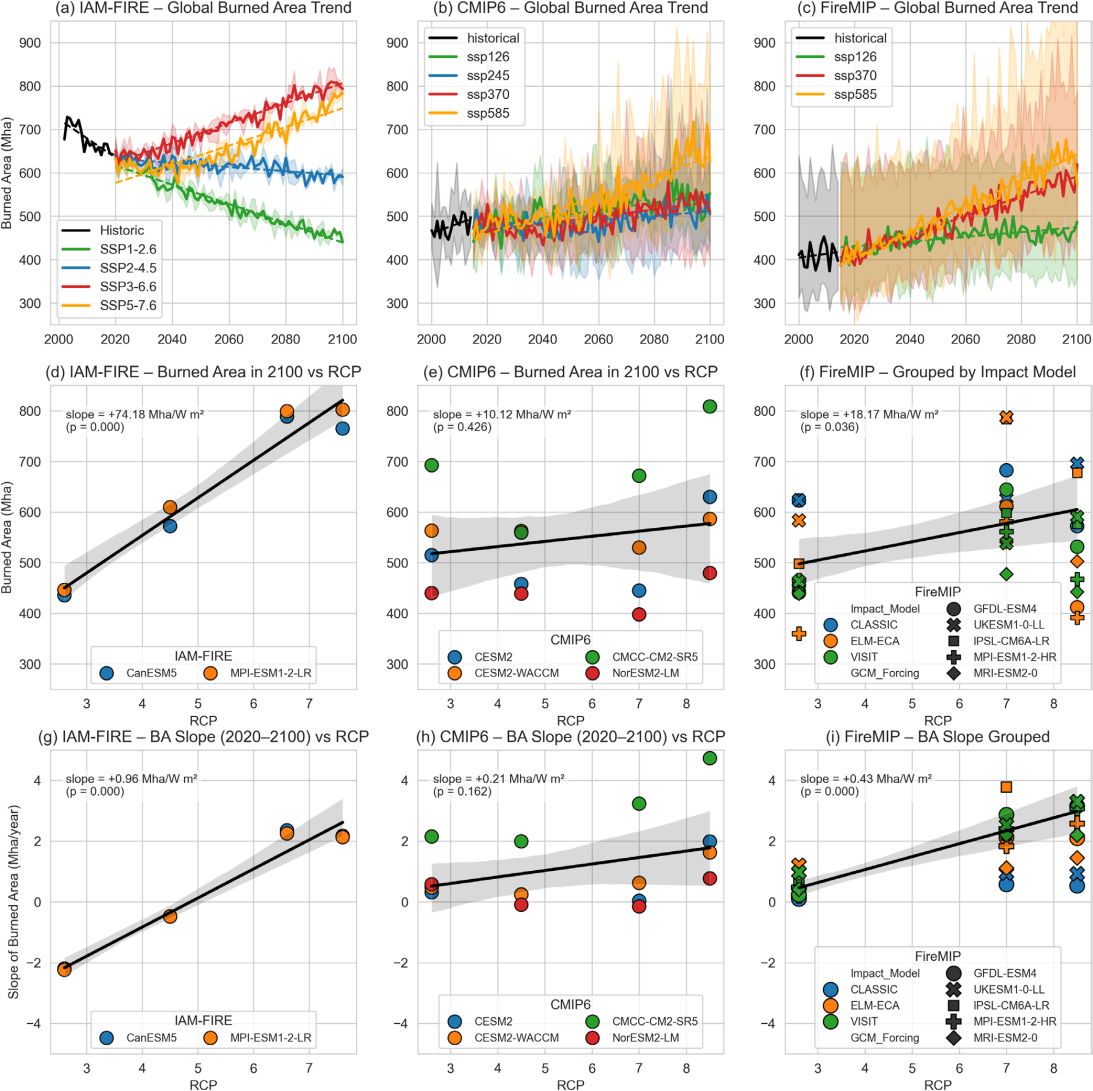
Comparison of time series from 2020 to 2100 for IAM‐FIRE (a), CMIP6 (b) and FireMIP (c) projections, of the relationship between BA in 2100 and RCP levels for IAM‐FIRE (d), CMIP6 (e) and FireMIP (f) and of the relationship between BA slope over 2020–2100 and RCP levels IAM‐FIRE (g), CMIP6 (h) and FireMIP (i). (a–c) The shaded areas represent the range around the mean of the 2 ESMs (CanESM and MPI) for IAM‐FIRE and the standard deviation for the 4 ESMs covered for CMIP6 (CESM2, CESM2‐WACCM, CMCC‐ESM2, Nor‐ESM2‐LM) and 3 fire impact models for FireMIP (CLASSIC, ELM‐ECA, VISIT).

During the historical period (2002–2019 for IAM‐FIRE, 2002–2015 for CMIP6 and FireMIP), global BA simulated by process‐based models is substantially lower than those of IAM‐FIRE, which is calibrated against GFEDv5 observations (695 Mha year^−1^ for IAM‐FIRE against 477 and 410 Mha year^−1^ for CMIP6 and FireMIP, respectively) (Figure 9a–c). The two ensembles also exhibit opposite historical trends: IAM‐FIRE reproduces the observed decline in global BA (−4.81 Mha year^−2^), while CMIP6 and FireMIP indicate weak increasing trends (+2.23 and +1.76 Mha year^−2^ respectively).

Over the projected period (2020–2100), process‐based models suggest a modest rise in total BA, ranging from +0.47 Mha year^−2^ under SSP1‐2.6 to +2.99 Mha year^−2^ under SSP5‐8.5 in FireMIP (CMIP6 exhibits less divergence with values comprised between 0.59 to 2.22 Mha year^−2^). IAM‐FIRE projects diverging trends ranging from −2.22 Mha year^−2^ (SSP1‐2.6) to 2.3 Mha year^−2^ (SSP3‐3.6) (Figure 9a–c). Fire suppressing mechanisms are thus significantly stronger in IAM‐FIRE than in process‐based models at low and medium levels of emissions—mostly through the inclusion of HDI non‐linear effects—which ultimately demonstrate the large structural uncertainties (cross‐model) associated with future drivers of wildfires.

In addition, IAM‐FIRE simulations show a strong and statistically significant sensitivity to radiative forcing (RCP level), while CMIP6 displays a weaker and non‐significant response and FireMIP a weaker but significant response (Figure 9d–f). In IAM‐FIRE, total BA in 2100 increases by approximately +74 Mha per W m^−2^ of forcing, and the interannual BA trend strengthens by +0.96 Mha year^−1^ per W m^−2^. In contrast, the CMIP6 and FireMIP ensemble exhibit much lower sensitivities (+10 and +18 Mha per W m^−2^, respectively), with significant trends across FireMIP projections only. These findings underscore the critical role of climate forcing in intensifying wildfire risk, with IAM‐FIRE indicating a more pronounced and robust increase in fire activity under stronger forcing.

## 4 Discussion

We presented BA and CE outputs from the novel IAM‐FIRE modelling framework. This section elaborates on the insights and limitations of the proposed framework in relation to previous studies while outlining potential avenues for future research.

### 4.1 Implications for Modelling Fire Impacts and Risks

#### 4.1.1 Historic Prediction of Fire Impacts

IAM‐FIRE reproduces observed totals and temporal trends of global BA and CE for both total and forest fires over the 2002–2019 period. Overall global performance is good and superior to similar GLMs: *R* ^2^ = 0.75 in this study against *R* ^2^ = 0.69 in Haas et al. (2022) and *R* ^2^ = 0.57 in Kavhu et al. (2025). Nonetheless, regional discrepancies remain. In the SHAS region, IAM‐FIRE projects higher values possibly due to limitations in modelling fuel and soil moisture and the current representation of suppressing effects from livestock grazing (assumed constant). Historically, deliberate and planned pastoral burning of grasslands and pastures was associated with positive residuals in previous GLMs, contributing to higher BA in regions like the Brazilian Cerrado and Caatinga (Perkins, Haas, and Millington 2025). In contrast, the lower values of CE in boreal regions may be caused by the high short‐term interannual variability of boreal fires since extreme fires with higher‐than‐average fuel consumption cannot be adequately captured by a statistical model focused on mean long‐term rates of fire drivers (Chuvieco et al. 2021; Haas, Prentice, and Harrison 2024).

#### 4.1.2 Role of Climate Change Through Climate and Vegetation

IAM‐FIRE captures the impacts of climate change on future BA and CE in two ways: (1) changing climatic conditions make fuel drier and more flammable, and (2) higher GHG concentrations increase fuel load through CO_2_ fertilization (e.g., GPP). Our study confirms that these two mechanisms could substantially increase global BA, particularly in high emission scenarios. Among regions, South America appears to be the most sensitive to changes in predictor signals, confirming the region's vulnerability to climate‐driven fire risk captured through fire weather projections (Quilcaille et al. 2023). Non‐tropical regions, particularly boreal North America and Europe, are also highly sensitive to climate change. Despite large uncertainties of ESM outputs, our results align with regional analyses that predict higher fire impacts due to fire weather even at lower latitudes, such as the Mediterranean (El Garroussi et al. 2024). Our results also show that CO_2_ fertilization increases both fuel loads and BA by enhancing plant growth (GPP), although these effects could be limited by nutrient availability (Kou‐Giesbrecht et al. 2025; Terrer et al. 2019). Despite the assumed constant fAPAR, this mechanism highlights the potential of fuel load control, such as prescribed burning or grazing, as effective fire management strategies (Perkins, Haas, and Millington 2025; Puig‐Gironès et al. 2025). Importantly, the pyrealm package is currently working on the development of a predictive fAPAR scheme implying that the effect of changing fAPAR could be accounted for in future versions of the P‐Model. This would allow fAPAR projections to be constructed based solely on climate and CO_2_ inputs (Zhou et al. 2025), providing a flexible and adaptable framework in future developments.

#### 4.1.3 Role of Socio‐Economic Development and Land Use Change

While human factors can significantly modulate fire regimes and override climate‐driven effects (Fernández‐García et al. 2023), fire models often represent them simplistically—through functions based on population density or cropland cover—and tend to disagree on their relative impacts (Haas, Keeping, et al. 2024; Perkins, Haas, Kasoar, et al. 2025). In contrast, IAM‐FIRE incorporates human influence through both HDI and land conversions. HDI emerges as a dominant factor, highlighting that future socio‐economic development can counteract climate‐driven impacts on fire regimes. This has been observed historically (Burton et al. 2024) and is also confirmed by the historic factorial decomposition of this study. It also aligns with recent studies showing that higher development levels reduce wildfire economic damages (Hwong et al. 2025) and are associated with more intensive fire suppression (Perkins, Haas, Kasoar, et al. 2025). Still, the non‐linear relationship shows that human development is effective only up to a certain point beyond which it cannot further suppress BA—perhaps due to suppression‐driven fuel accumulation and fire intensity. Strong fire suppression programs in highly‐developed countries could indeed lead to rebound effects through excessive management, leading to accumulation of fuel and increased likelihoods of extreme fires (Kreider et al. 2024). In addition, the SSP framework does not account for climate impacts and hence HDI projections could be overestimated, especially in the high‐emission scenarios. These projections also have equity implications as unequal HDI developments could lead to different readiness to manage fire across regions (Hwong et al. 2025). While they remain challenging to model, future extreme fires may further dampen human‐driven suppressing effects and hence increase total BA projections, particularly in northern latitudes with worsening fire weather (Hantson et al. 2024).

LULUC also plays a significant role, having a varying effect on fires depending on the extent of grassland conversion versus cropland expansion. Grassland is the largest predictor of the GLM as herbaceous plants and shrubs are the dominant vegetation that burn globally, mostly located in Africa (Chen et al. 2023a). Grazing pressure has a mitigating effect on BAs by reducing fuel load, consistent with the literature (Andela et al. 2017). Our results confirm that LULUC effects are particularly pronounced in regions with high projected cropland expansion under SSP2 and SSP3, such as the USA and Europe. While IAM‐FIRE does not cover anthropogenic fire types like agricultural burning or deforestation fires and considering that a simple proxy of fragmentation was proved to not be influential in the GLM (Table S9), the results still illustrate that LULUC can have divergent impacts across ecosystems. The representation of these effects—which also depend on ecosystems' adaptation to fires (Harrison et al. 2021)—should be improved in fire models and combined with explicit representation of fragmentation effect (Bowring et al. 2024; Driscoll et al. 2021; Rosan et al. 2022).

#### 4.1.4 Impacts of Fire on the Carbon Cycle

Our study suggests that FC is expected to continue increasing in the future, in line with the trends observed in recent decades (Jones, Veraverbeke, et al. 2024). IAM‐FIRE projects an increase in FC driven by fuel dryness, but does not fully account for growth in fuel load. Still, observations from 2010 to 2019 report a positive 10‐year trend in fuel load, with an overall increase of +4.5 Pg year^−1^ (McNorton and Di Giuseppe 2024). As a result, our estimates of FC may still be conservative, particularly in scenarios with high CO_2_ fertilization that enhance fuel accumulation.

In terms of CE, global carbon loss from fire can lead to shifts in vegetation cover and changes in carbon storage (Lasslop et al. 2020). IAM‐FIRE does not explicitly model post‐fire carbon sinks from regrowth and only provides gross CE. Warming and human development trajectories strongly determine whether future fire regimes reduce or enhance vegetation carbon stocks. While post‐fire ecosystems can act as carbon sinks over decades, partially offsetting immediate emissions, ecosystem degradation may slow or prevent full recovery (Pereira et al. 2024; Zheng et al. 2021) and hence generate foregone emissions (Maxwell et al. 2019). Accounting for these dynamics would be required to assess the net impact of future fire regimes on the climate.

There are important caveats regarding recently observed fire activity considering that our calibration period does not include 2020–2025. Recent fire‐related CE increased strongly due to climate change despite below‐average BA, according to attribution analyses from the State of Wildfires 2024–2025 (Kelley et al. 2025). While IAM‐FIRE shows a decoupling between BA and CE in some scenarios and regions, the rapid acceleration of climate change in recent years suggests that projections may underestimate actual impacts (Potapov et al. 2025). By contrast, it is also possible that current boreal fires are secular events—the unique product of rapid warming after long‐term fuel accumulation—and that future fire activity in the region will be limited by slow post‐fire vegetation regrowth (Tepley et al. 2025). Overall, such complex climate‐vegetation feedbacks are an inherent uncertainty in projecting future fire regimes, requiring detailed process‐based ecosystem modelling beyond the scope of this study (Hantson et al. 2024).

### 4.2 Comparison With Literature and Process‐Based Models

Estimations of fire impacts vary widely across the literature, largely due to differences in benchmark datasets, predictor selection, and modelling frameworks. IAM‐FIRE is parameterized with GFEDv5 which captures small fires more efficiently than previous datasets, resulting in significantly higher BA estimates than other lower‐resolution BA datasets (Chen et al. 2023a). While CMIP6 and FireMIP models generally report a positive historical BA trend, our findings align with studies indicating an overall decline in global BA. For instance, Wu et al. (2021) found a decreasing BA at a rate of −4.85 Mha year^−2^ over the period 2000–2013 using a process‐based global fire model (SEVER‐FIRE), which is consistent with IAM‐FIRE estimates of −4.81 Mha year^−2^ over 2002–2019. GFEDv5 confirms that the decline observed in 1997–2015 (Andela et al. 2017) has continued in the 2016–2020 period (Chen et al. 2023a). For future periods, Wu et al. (2021) reports increasing BA only in the scenario with high emissions, rapid population growth, and rapid urbanization (S4 with RCP 8.5), similarly to IAM‐FIRE which reports increasing BA with high emissions and low human development (SSP3‐6.6). A recent study also found diverging trends between low and high mitigation scenarios, highlighting the importance of human activities in reducing BAs through fragmentation and infrastructure (Haas, Prentice, and Harrison 2024).

The GFEDv5 global carbon emissions (2.9 Pg C year^−1^) are approximately 50% higher than those in GFED4s and closely aligned with IAM‐FIRE's estimate of 2.86 Pg C year^−1^, which explains the gap with CMIP6 and FireMIP in historic periods. Major uncertainties also persist in FC modelling, primarily due to the high natural variability of combustion rates and inconsistencies among fuel load datasets. Recent evidence suggests that forest fire emissions have increased by 60% globally during 2001–2022 (Jones, Adloff, et al. 2024) while unprecedented high forest fires were observed in 2023 and 2024 (Kelley et al. 2025; Potapov et al. 2025). This contrasts with IAM‐FIRE results and illustrate the importance of expanding the GLM's temporal coverage amid recent surges in global fire activity. For future periods, Park et al. (2023) found that global fire CE decreased in scenarios including for SSP3‐6.0 and SSP3‐8.5 due to increases in GDP per capita. While this is aligned with the strong mitigating effect of HDI improvements—which is partially derived from GDP—reported here, unlike in Park et al. (2023), IAM‐FIRE projects meteorological and vegetation‐based fire enhancement in high emission scenarios that significantly overweight suppressing socioeconomic factors, resulting in increasing global CE.

### 4.3 Methodological Insights and Limitations

A key contribution of this study is the use of the novel STITCHES climate emulator, which offers advantages relative to using standard scenarios run by ESMs included in CMIP6. First, STITCHES allows the analysis of GCAM projections using SSP5 and SSP3 socio‐economic assumptions while reaching two radiative forcing levels, 7.6 and 6.6, respectively, which would not be available from off‐the‐shelf climate model experiments in the CMIP6 archive. Thus it enables a strictly consistent representation of climate impacts in IAMs for forcing levels more closely aligned with current expectations for high‐emission scenarios (Burgess et al. 2020). Second, compared to ESMs that can take days or weeks to run, STITCHES and the BASD process run in only a few hours, reducing the computational cost. This modelling framework can further enable impact studies (assessed by WGII) based on climate projections (assessed by WGI) while reflecting the latest socio‐economic trajectories (assessed in WGIII), thus supporting a more integrated approach across the three IPCC WGs. Third, unlike most emulators, STITCHES can emulate daily‐scale variables, which is essential for including NDD and precipitation rolling sum predictors. However, STITCHES as a SCE has notable constraints that should be acknowledged. The time slicing approach hinders the emulation of path‐dependent quantities and impacts lasting over a decade (Tebaldi et al. 2022). Relatedly, STITCHES cannot emulate temperature trajectories that are not well represented in the ESM archive—such as the increasingly relevant overshoot pathways which were not largely modelled in CMIP6 beyond mild overshoot in SSP1‐2.6. Emulating well represented trajectories is also constrained by the availability of ESM outputs including all required variables at sufficient temporal resolution, which can limit the set of usable simulations in existing archives. The need to emulate 4 variables at daily scale in this study has limited the number of ESMs that could be emulated (CanESM5 and MPI) while a higher number would be desirable to better account for structural uncertainties between ESMs.

At the core of IAM‐FIRE, the GLM predicts total BA fraction using an empirical approach that contrasts with the process‐based fire modules of ESMs. The empirical setup allows flexible testing of different predictor combinations, interactions and non‐linearities, facilitating model intercomparison and sensitivity analyses. It is also computationally efficient, which, when combined with STITCHES, enables seamless integration into IAMs without the heavy overhead associated with fire‐enabled ESMs. Empirical models, however, come with trade‐offs. Because they rely on historical data, they may fail to predict novel fire regimes not represented in past observations (Abalo et al. 2025). Emerging phenomena such as boreal (Corning et al. 2024; Hanes et al. 2025) or Amazonian (Bourgoin et al. 2025; Le Page et al. 2017; Pereira et al. 2024) extreme fires may therefore be underestimated. Moreover, empirical relationships implicitly assume stationarity (i.e., the links between drivers and impacts remain constant), which may not hold under strong climate forcing (Hwong et al. 2025). While GLMs have demonstrated reasonable skill in out‐of‐sample tests, caution is warranted when applying them to climates outside the historical envelope (Perkins, Haas, Kasoar, et al. 2025).

IAM‐FIRE employs a process‐based methodology to derive secondary variables from total BA. This introduces error propagation: moderate biases in total BA can lead to significant deviations from GFEDv5 benchmarks in regional forest CE (e.g., in SHAS and in boreal regions). IAM‐FIRE does not currently differentiate between managed and unmanaged forest fires, does not cover peatland, agricultural, and deforestation fires and omits fire characteristics like fire size and intensity (Haas et al. 2022; He et al. 2025). Although the framework is optimized for wildfire dynamics, further disaggregation could expand its applicability to studies of LULUC, carbon feedbacks, and emissions accounting. Similarly, IAM‐FIRE computes fire impacts at annual scale. Fires are inherently seasonal processes and some studies recommend the inclusion of sub‐daily data for meteorological drivers (Matteo et al. 2025). This temporal aggregation limits the framework's precision for applications involving economic losses or air quality impacts sensitive to intra‐seasonal fire variability.

### 4.4 Future Work

Future research could focus both on improvements and applications of IAM‐FIRE. Improvements should address key gaps in model representation and scope, for instance, to incorporate missing drivers and to extend the historical period to include recent years of extreme fire activity. Benchmarking against alternative modelling approaches could strengthen predictive performance. Moreover, refining spatial resolution through land use (Forrest et al. 2024) or regional disaggregation (Jones, Veraverbeke, et al. 2024) and expanding the climate emulation capabilities could improve robustness and reduce structural uncertainties across global fire projections.

Future applications of IAM‐FIRE should expand its scenario coverage, assess broader fire impacts, and enhance integration of feedbacks in IAMs. As for scenario coverage, the new variants produced in the context of ScenarioMIP for the next IPCC assessment report (Dunne et al. 2025; van Vuuren et al. 2026) should be explored, as these will presumably yield different LULUC projections and land‐based mitigation options, with potential justice and fairness implications (Zimm et al. 2024). As a computationally agile emulator, IAM‐FIRE can be useful to characterize the effects of fire risk on these land‐use trajectories at a small fraction of the substantial cost implied by running full ESMs. Furthermore, IAM‐FIRE can be used to quantify the economic (Hwong et al. 2025), ecological (Lasslop et al. 2020), and health (Alari et al. 2025; Zhao et al. 2025) consequences of fires which would strengthen its role in interdisciplinary assessments of future climate risks.

While IAM‐FIRE demonstrates the potential of combining climate emulators and empirical models for rapid, transparent, and flexible impact assessments, incorporating fire‐driven feedbacks between land and climate systems within IAMs would be needed to ultimately improve the understanding of land‐based mitigation and adaptation dynamics (Jäger et al. 2024; Luo et al. 2025). At this stage, IAM‐FIRE only produces gross fire emissions while a full assessment of fire‐climate feedbacks would require net emissions, which depend on post‐fire vegetation regrowth, legacy carbon dynamics, and additional biophysical effects such as aerosols and albedo. Capturing these dynamics is a key priority for future work to enable a complete evaluation of fire‐induced climate feedbacks. In particular, these developments would pave the way towards dynamic representations of carbon densities driven by novel fire regimes and fire‐responsive land allocation decisions within the IAM framework that account for different sources of uncertainty in climate projections (Wu et al. 2022). IAM‐FIRE could also improve how fire emissions are modelled in Hector since these are not explicitly captured in the estimate of the total carbon flux from atmosphere to land. In this way, IAM‐FIRE could become an instrumental tool to support more internally consistent assessments of land‐based mitigation and climate risks—an essential step toward reducing uncertainties in next‐generation IAMs and CMIP7 frameworks.

## Author Contributions

**Théo Rouhette:** writing – original draft, writing – review and editing, conceptualization, methodology, investigation, formal analysis, visualization, software, data curation. **Claudia Tebaldi:** methodology, software, writing – review and editing. **Dirk‐Jan Van de Ven:** supervision, funding acquisition, writing – review and editing, validation, conceptualization. **Neus Escobar:** writing – review and editing, supervision, funding acquisition, validation, conceptualization. **Kanishka Narayan:** software, methodology, writing – review and editing. **Oliver Perkins:** writing – review and editing, investigation, methodology, formal analysis. **Olivia Haas:** methodology, writing – review and editing, investigation, formal analysis.

## Funding

This work was supported by HORIZON EUROPE Framework Programme (101184374 and 101081179), Leverhulme Trust (RC‐2018‐023), María de Maeztu (CEX2021‐001201‐M), Eusko Jaurlaritza.

## Conflicts of Interest

The authors declare no conflicts of interest.

## Supporting information


**Figure S1:** Map of land use fractions in the ESA basemap used to downscaled GCAM projections with Demeter.
**Figure S2:** Forest Proportion of Total BA used in the proportion approach based on GFEDv5.1.
**Figure S3:** Fuel consumption (gC.m2 burned) derived from McNorton and Di Giuseppe (2024) fuel load datasets and CC factors from van Wees et al. (2022) and used as derive dynamic changes FC in simulated period considering VPD scaling on CC.
**Figure S4:** Fuel consumption (gC.m2 burned) derived from GFEDv5 and used as basemap to project dynamic changes to FC in simulated period.
**Figure S5:** Temperature trajectory (left) and CO2 concentration (right) of the SSP‐RCP scenarios modelled by GCAM & Hector.
**Figure S6:** Annual values for 2003 of observed and observed response variables and mean latitudinal distributions over historic period (2002–2019) for total BA (a–c), forest BA (d–f), total CE (g–i), and forest CE (j–l). Maps for BA are shown in fraction (0–1) and latitudinal bands in Mha. Carbon emissions are reported in TgC.
**Figure S7:** Annual values for 2016 of observed and observed response variables and mean latitudinal distributions over historic period (2002–2019) for total BA (a–c), forest BA (d–f), total CE (g–i), and forest CE (j–l). Maps for BA are shown in fraction (0–1) and latitudinal bands in Mha. Carbon emissions are reported in TgC.
**Figure S8:** Regional trends for total burned areas for observations and predictions from 2002 to 2019.
**Figure S9:** Regional trends for forest burned areas for observations and predictions from 2002 to 2019.
**Figure S10:** Partial residual plots of the predictor variables of the final GLM. Colours correspond to the category (vegetation, topography, land uses, climate and socio‐economics).
**Figure S11:** Sensitivity analysis of GPP estimates using default fAPAR from default historical period (2018–2019) versus past period (1983–2001). Left panel represents average spatial difference and right panel represent the temporal trend of the global sum of GPP estimates.
**Figure S12:** Global BA trends for historic period (2002–2019) for observation data (GFED5), final GLM with default GPP, and modified GLM with GPP based on past fAPAR.
**Figure S13:** Total burned area per GFED regions.
**Figure S14:** Forest burned area per GFED regions.
**Figure S15:** Total carbon emissions per GFED regions.
**Figure S16:** Forest carbon emissions per GFED regions.
**Figure S17:** Trends of the drivers of total burned area.
**Figure S18:** Factorial analysis of drivers per GFED regions. Error bars represent the range between the two ESMs.
**Figure S19:** Factorial decomposition of limiting drivers for global scenarios. Error bars represent the range between the two ESMs.
**Figure S20:** Factorial analysis of limiting factors per GFED regions. Error bars represent the range between the two ESMs.
**Table S1:** Description of the ESMs emulated by STITCHES in the IAM‐FIRE framework.
**Table S2:** Treatment order for the Demeter application with the ESA basemap.
**Table S3:** Transition allocation for the Demeter application with the ESA basemap.
**Table S4:** Combustion completeness factors per biome and fuel types.
**Table S5:** Factorial decomposition runs for limiting factors and drivers. A prefix “+” means the group of variables is varying/dynamic while a prefix “−” means it is fixed at the 2002‐value for the historic period and at the 2020‐value for future period.
**Table S6:** Summary statistics of the GAMS used to identify non‐linear relationships between predictors and BA.
**Table S7:** Summary statistics of the final GLM model used to predict burned area. Predictors included were all significant at Pr (>|*t*|) < 1.97e−11. VPD: Vapor Pressure Deficit, NDD: Number of Dry Days, GPP: Gross Primary Productivity, VRM: Vector ruggedness measure, HDI: Human Development Index.
**Table S8:** Summary statistics of the rolling hindcast results for out‐of‐sample validity checks.
**Table S9:** Summary statistics of the modified GLMs.
**Table S10:** Results of GLM sensitivity analysis using GPP estimates based on past fAPAR (1983–2001).

## Data Availability

The Global Change Analysis Model (GCAM) version 8.2 is an open‐source model that can be downloaded at https://www.github.com/JGCRI/gcam‐core. GFEDv5 data was obtained from https://doi.org/10.5281/zenodo.16794692. W5E5v2.0 data web obtained from the ISIMIP repository at https://data.isimip.org/10.48364/ISIMIP.342217. ESA CCI Plant Functional Types Products was obtained from the CEDA archive at https://catalogue.ceda.ac.uk/uuid/26a0f46c95ee4c29b5c650b129aab788/. The ECMWF Fuel Characteristics V1 was obtained from the Copernicus Climate Data Store at cems_fuelmodel_data@aux.ecmwf.int. *Code Availability Statement*: The source code and instructions to run IAM‐FIRE can be found in the online repository https://github.com/bc3LC/IAM‐FIRE and input are provided in Zenodo https://doi.org/10.5281/zenodo.20312037. The source code used for the data analysis and the figure generation, both for the manuscript and the Supporting Information, can be found in the online repository https://github.com/LinoHub/IAM‐FIRE_projections/ and https://zenodo.org/records/20311852.
