# Supplementary material for: IAM‐FIRE: A Climate Emulator–Based Framework to Project Wildfire Impacts and Risks for Integrated Assessment Models
Source: Glob Chang Biol. 2026 Jun 18;32(6):e70951. doi: 10.1111/gcb.70951 (PMC13280204; doi:10.1111/gcb.70951)
Supplement: Supplementary file 1 — Figure S1: Map of land use fractions in the ESA basemap used to downscaled GCAM projections with Demeter. Figure S2: Forest Proportion of Total BA used in the proportion approach based on GFEDv5.1. Figure S3: Fuel consumption (gC.m2 burned) derived from McNorton and Di Giuseppe (2024) fuel load datasets and CC factors from van Wees et al. (2022) and used as derive dynamic changes FC in simulated period considering VPD scaling on CC. Figure S4: Fuel consumption (gC.m2 burned) derived from GFEDv5 and used as basemap to project dynamic changes to FC in simulated period. Figure S5: Temperature trajectory (left) and CO2 concentration (right) of the SSP‐RCP scenarios modelled by GCAM & Hector. Figure S6: Annual values for 2003 of observed and observed response variables and mean latitudinal distributions over historic period (2002–2019) for total BA (a–c), forest BA (d–f), total CE (g–i), and forest CE (j–l). Maps for BA are shown in fraction (0–1) and latitudinal bands in Mha. Carbon emissions are reported in TgC. Figure S7: Annual values for 2016 of observed and observed response variables and mean latitudinal distributions over historic period (2002–2019) for total BA (a–c), forest BA (d–f), total CE (g–i), and forest CE (j–l). Maps for BA are shown in fraction (0–1) and latitudinal bands in Mha. Carbon emissions are reported in TgC. Figure S8: Regional trends for total burned areas for observations and predictions from 2002 to 2019. Figure S9: Regional trends for forest burned areas for observations and predictions from 2002 to 2019. Figure S10: Partial residual plots of the predictor variables of the final GLM. Colours correspond to the category (vegetation, topography, land uses, climate and socio‐economics). Figure S11: Sensitivity analysis of GPP estimates using default fAPAR from default historical period (2018–2019) versus past period (1983–2001). Left panel represents average spatial difference and right panel represent the temporal trend of the global sum of G [file GCB-32-e70951-s001.pdf]

Supplementary Material of “IAM-FIRE: a Climate Emulator–Based Framework to Project Wildfire Impacts and Risks for Integrated Assessment Models”

Running title

Emulator-based Projections of Fire Impacts

Authors

Rouhette, Théo[1,2]. Van de Ven, Dirk-Jan[1]. Narayan, Kanishka[3]. Tebaldi, Claudia[4]. Perkins, Oliver[5,6,7]. Haas, Olivia[5,8], Escobar, Neus[1,9]

[1] Basque Centre for Climate Change (BC3), Scientific Campus of the University of the Basque Country, Leioa, Spain

[2] Institute of Environmental Science and Technology (ICTA), Universitat Autònoma de Barcelona, Cerdanyola del Vallès, Spain

[3] Joint Global Change Research Institute (JGCRI), Pacific Northwest National Laboratory (PNNL), and University of Maryland, College Park, MD 20740, USA

[4] Earth System Science Interdisciplinary Center, University of Maryland, College Park, Maryland, USA

[5] The Leverhulme Centre for Wildfires, Environment, and Society, Imperial College London, SB7 2BX, London, UK

[6] Department of Geography, King’s College London, WC2B 4BG, London, UK

[7] School of Environmental Sciences, University of East Anglia, Norwich, NR4 7TJ, UK

[8] Geography and Environmental Science, University of Reading, Reading, UK

[9] Biodiversity and Natural Resources (BNR) Program, International Institute for Applied Systems Analysis (IIASA), Laxenburg, Austria

Corresponding author

Théo Rouhette – theo.rouhette@bc3research.org

Keywords

Burned area; Climate change; Climate emulators; Earth System Models; Integrated Assessment Models; Fire carbon emissions; Socioeconomic development; Wildfires.

Table of abbreviations

Abbreviation	Definition
BA	Burned area
CE	Carbon emissions
CMIP6	Coupled Model Intercomparison Project Phase 6
ESM	Earth System Model
FC	Fuel consumption
GCAM	Global Change Analysis Model
GFED	Global Fire Emissions Database
GLM	Generalized Linear Model
GPP	Gross primary productivity
HDI	Human development index
IAM	Integrated assessment model
NDD	Number of dry days
SCE	Spatial climate emulator
SSP	Shared Socioeconomic Pathway
TPI	Topographic position index
VPD	Vapor pressure deficit
VRM	Vector ruggedness measure

1. Supplementary Material: Methods

1.1. GCAM & Hector

In this study we use the release of GCAM v8.2 which updates the baseline year from 2015 to 2021. GCAM is a global integrated assessment model that simulates interactions between five key systems: energy, water, land, climate, and the economy. It operates in 5-year time steps up to 2100, analysing how policy and technology assumptions affect emissions and resources. Hector v3.2.0 is the default climate model within GCAM. Currently the GCAM sectors interact with Hector via emissions. At every time step, emissions from GCAM are passed to Hector. Hector converts these emissions to concentrations when necessary, and calculates the associated radiative forcing, as well as the response of the climate system and earth system (e.g., temperature, carbon-fluxes, etc.). Key climate metrics for v3.2.0, reviewed in Dorheim et al., (2024), include an equilibrium climate sensitivity (ECS) of 3, a transient climate response to cumulative carbon emissions (TCRE) of 1.51, and a transient climate response (TCR) of 1.84.

1.2. IAM-FIRE

1.2.1. Climate emulator: STITCHES

We ran the Spatial Climate Emulator (SCE) STITCHES which emulates output variables of a target Earth System Model (ESM) using CMIP6 data by connecting together short segments of existing simulations from the target ESM. Each emulation by STITCHES targets one ESM only and uses the CMIP6 experiments from the ESM as building blocks which are matched to the target scenario's global temperature trajectory.

In this study we use STITCHES to emulate output variables for 2 Earth System Models (ESMs): CanESM5 and MPI-ESM1-2-LR whose descriptions are provided in Table S1. The outputs from STITCHES are then processed through the BASD package provided by the GCAM modelling ecosystem.

Table S1. Description of the ESMs emulated by STITCHES in the IAM-FIRE framework

**Earth System Model (ESM)**	**CanESM5**	**MPI-ESM1-2-LR**
Institution	Canadian Centre for Climate Modelling and Analysis (CCCma), Environment and Climate Change Canada	Max Planck Institute for Meteorology (MPI-M)
Atmospheric Model	CanAM5	ECHAM6.3
Model Sensitivity	High equilibrium climate sensitivity (~5.62 K)	Moderate equilibrium climate sensitivity (~2.8 K)
Land Model	CLASS3.6/CTEM1.2	JSBACH3.20
Resolution	2.8° atm.; 1.0° ocean	1.8° atm.; 1.5° ocean
Key references	(Swart et al., 2019)	(Gutjahr et al., 2019)

1.2.2. Vegetation projection: P-Model

The P-Model requires several variables and parameters to predict Gross Primary Productivity (GPP). In this framework we assume a constant fraction (0.5) of incoming solar irradiance that is photosynthetically active radiation (PAR) to convert solar radiation (RSDS) into photosynthetic photon flux density (PPFD). We also assume a fixed spatially-explicit monthly fAPAR over the projected period based on average climatology of historical values from GIMMS FPAR4g (W. Zhao et al., 2024). The seasonality of GPP is estimated with Eq. 1 as follows:

$$GPP_{seasonality} = \frac{max(GPP_{monthly}) - min(GPP_{monthly})}{mean(GPP_{monthly})} \quad \textbf{(Eq. 1)}$$

1.2.3. Land use downscaling: Demeter

To ensure harmonized land use projections between the historic data used for the GLM and the simulated period, we harmonize GCAM projections to ESA land cover classification prior to running Demeter. The procedure adjusts the area of each land use type in the GCAM projections at the starting point (governed by the ESA base map, i.e., in 2015) to match the base map but keep the fractional change of each land use type as projected by GCAM in the following time steps. The complete description is provided in Chen et al., (2020).

This procedure produces a harmonized GCAM projections to be used in Demeter based on the ESA basemap. The average land use fractions from ESA CCI product are shown in Figure S1. The downscaling tool Demeter provides spatially-explicit land use projections from GCAM aggregated land use. The parameters include (1) the treatment order, which determines the sequence of land cover type allocations; (2) the transition priority, which establishes the hierarchy of land cover transitions; and (3) the spatial constraints, such as soil workability and nutrient availability distributions. The treatment order and the transition priority were modified to match the new ESA basemap (Table S2 and S3) while the spatial constraints were implemented as in default mode but with the 0.5-deg resolution of the ESA basemap.

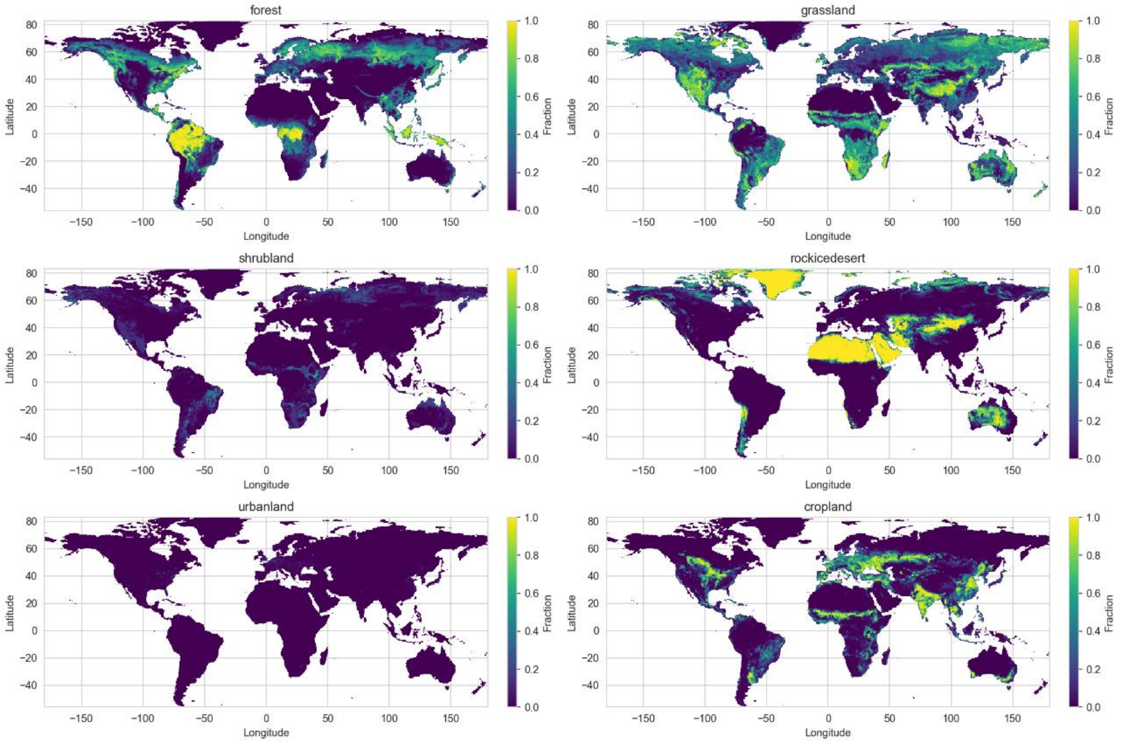

Figure S1. Map of land use fractions in the ESA basemap used to downscaled GCAM projections with Demeter

Table S2. Treatment order for the Demeter application with the ESA basemap

Land Use	Order
Cropland	1
Grassland	2
Forest	3
Shrubland	4
Urbanland	5
RockIceDesert	6

Table S3. Transition allocation for the Demeter application with the ESA basemap

Land Use	Urbanland	RockIceDesert	Cropland	Forest	Grassland	Shrubland
Urbanland	0	5	2	1	3	4
RockIceDesert	5	0	1	2	4	3
Cropland	4	5	0	3	1	2
Forest	4	5	1	0	2	3
Grassland	4	5	3	1	0	2
Shrubland	4	5	1	2	3	0

1.2.4. Statistical modelling of burned areas: GLM

This section provides details on the computation of the independent variables of the GLM. To represent climatic drivers, we estimated vapor pressure deficit (VPD), number of dry days (NDD), wind speed and 30-day precipitation rolling sum as follows:

1. **Vapor Pressure Deficit (VPD).** VPD is a critical indicator of atmospheric demand for water vapor which is projected to increase under global warming (Fang et al., 2022; Williams et al., 2024) and is associated with increased forest fire risks (Clarke et al., 2022). VPD is estimated following the equations of Bjarke et al., (2023), which first estimates the saturated vapor pressure e_s using air temperature (Eq. 2) and then VPD using e_s and relative humidity h_r (Eq. 3):

$$e_s = 0.6108^{\frac{17.27*TAS_{avg}}{TAS_{avg}+237.3}} \quad \textbf{(Eq. 2)}$$

$$VPD = \left(1 - \frac{h_r}{100}\right) * e_s \quad \textbf{(Eq. 3)}$$

2. **Number of Dry Days (NDD).** NDD is used as a proxy for biomass production limitations, through which moisture availability affect the amount of combustible vegetation (Kavhu et al., 2024). The monthly number of dry days is estimated as the number of days with less than 1mm of precipitation. NDD seasonality is estimated to account for periods of wet vs dry seasons as in Eq. 1 with NDD inputs.
3. **Wind speed.** Wind speed is associated with fire spread, but was negatively related to burned areas in previous studies (Haas et al,. 2022).The wind speed value selected for a given year is the monthly value of the hottest month in that year.
4. **30-Day Precipitation Rolling sum**. The 30-day rolling sum of precipitation is used to quantify medium-term moisture accumulation in vegetation and soil, which can inhibit or facilitate fire ignition and spread. By summing precipitation over a moving 30-day window, this metric captures periods of sustained wetness or dryness that affect fuel availability and flammability. Unlike indices like the Fine Fuel Moisture Code (FFMC) are indeed designed to predict daily ignition probability and fire behaviour, this indicator reflects a "fire climatology" perspective that prioritizes landscape-scale fire suitability over the day-to-day weather-driven ignition physics.

For drivers related to vegetation and land uses, we use gross primary productivity (GPP) and fractions of grassland, shrubland, forestland and cropland.

1. **Gross Primary Productivity (GPP).** GPP is used as an index of vegetation productivity (Krause et al., 2022) and is derived from the P-Model. GPP seasonality is estimated to account for periods of high vs. low productivity as described above.
2. **Land use fractions.** The land use fractions are derived from the ESA CCI product for Plant Functional Types aggregated at 0.5-degree resolution by W. Li et al., (2018). The land uses covered are forest (PFT 1-4), grassland (PFT 9), shrubland (PFT 5-8) and cropland (PFT 10).

3. **Grazing pressure.** The grazing pressure is derived from (Perkins et al., 2025) and is a measure of the consumption of biomass per unit of area in a grid cell. The average over the 2001-2014 is computed from the raw data and applied as a static layer to the GLM. The layer is then held constant over the simulated period (2020-2100).

We represent the fire ignition and suppression through both topographic and socio-economic drivers. For the former, we use from Amatulli et al., (2018):

1. **Topographic Positioning Index (TPI).** TPI provides information on valleys (negative values) and ridges (positive values).
2. **Vector ruggedness measure (VRM).** VRM captures variability in slope and aspect into a single measure, providing a measure for terrain heterogeneity.

For the socio-economic variables, we use:

1. **Human Development Index (HDI).** HDI captures the country's social and economic development levels, providing a nuanced understanding of the socio-economic context shaping fire behavior (Cuaresma & Lutz, 2016; Liu et al., 2024).

The burned area and the predictors are regridded from their original resolution to 0.5-degree resolution using bilinear interpolation. From monthly data, we aggregate all the variables to an annual layer. Per year, for climatic drivers, we select the maximum monthly value of VPD and NDD, the average wind speed of the hottest month, and the average 30-day rolling sum for precipitation. For all other predictors, we compute the mean annual value. From a dataset spanning 2002-2019, we compute a mean/average layer to train the GLM.

We select a binomial GLM since the response variable (BA fraction) can be equated to the probability of burning ranging from 0 to 1. The expected burned area fraction μ is linked to the linear predictor using a logit link function (Eq. 4):

$$logit(\mu) = \log\left(\frac{\mu}{1-\mu}\right) \qquad \textbf{(Eq. 4)}$$

The initial GLM equation with the selected predictors is shown in Eq. 5:

$$logit(\mu) = \beta_0 + \beta_1 \log(VPD) + \beta_2 \log(NDD) + \beta_3 \log(NDD\_s) + \beta_4 \log(sfcWind) + \beta_5 \log(PR\_sum) + \beta_6 \log(GPP) + \beta_7 \mathrm{TPI} + \beta_8 VRM + \beta_9 HDI + \beta_{10} Grassland + \beta_{11} Cropland + \beta_{12} Shrubland + \beta_{13} Grazing\_pressure \qquad \textbf{(Eq. 5)}$$

Where β0 is the intercept term, β1- β13 are the beta coefficients associated with climatic, productivity, topographic, and land-use predictors (see details below for results and alternative GLMs).

Some variables are known for exhibiting non-linear relationship with burned area, such as VPD (Juang et al., 2022). For this reason, we test for non-linear relationship among the predictors using a Generative Additive Models (GAMs). We use the GAMs as an identification process to add polynomial terms to the GLM. We run the GLM and remove variables that are not significant or do not improve the model performance (detailed results are presented in Section 2.1.2). We reach the final equation:

$$logit(\mu) = \beta_0 + \beta_1 \log(VPD) + \beta_2 \log(NDD) + \beta_3 \log(NDD\_s) + \beta_4 \log(NDD_s^2) + \beta_5 \log(PR\_sum) + \beta_6 \log(GPP) + \beta_7 VRM + \beta_8 Grassland + \beta_9 Grassland^2 + \beta_{10} Cropland + \beta_{11} HDI + \beta_{12} HDI^2 + \beta_{13} Grazing\_pressure \qquad \textbf{(Eq. 6)}$$

To evaluate the model, we use the coefficient of determination (R2), the normalized mean error (NME) and root mean square error (RMSE). NME is estimated in Eq. 7 following Hantson et al., (2020):

$$NME = \frac{\sum A_i |obs_i - sim_i|}{\sum A_i |obs_i - \overline{obs}|} \quad \textbf{(Eq. 7)}$$

And the RMSE with Eq. 8 as follows:

$$RMSE = \sqrt{\sum(\overline{obs_i} - \overline{sim_i})^2} \quad \textbf{(Eq. 8)}$$

where *obs* stands for historic values and *sim* for predicted values are summed over all cells *i.*

To evaluate the predictive robustness and temporal generalizability of the GLM, we conducted a systematic rolling-window hindcast (temporal cross-validation). Unlike standard in-sample metrics, which may overstate performance through overfitting to the training period (2002–2019), this approach tests the model's ability to predict burned area (BA) for years it has not seen during the parameterization phase. We defined a sliding window of three consecutive years (e.g., 2002–2004, 2003–2005, ..., 2017–2019) to serve as the out-of-sample "test" set. For each iteration, the GLM was re-trained using all available data outside of that specific 3-year window. The frozen model coefficients were then applied to the climate and socio-economic drivers of the excluded years to generate predicted BA fractions.

1.2.5. Burned area disaggregation

In GFED5, the BA are disaggregated according to land cover type (LCT) from MODIS MCD12Q1 and then aggregated into 4 vegetation classes. The modified MODIS LCTs are derived from the vegetation maps by van Wees et al., (2022) which depart from the original MODIS product to fit the GFED5 requirements.

Here, we estimate the forest BA proportion from GFED5 BA data which ensures internal consistency compared to using the original MODIS mask. The forest and non-forest BA are estimated with Eq. 9-11 and Figure S2 shows the constant forest proportion computed from GFED5 and used throughout the simulated period.

$$Forest\ BA\ Proportion = \frac{Forest\ BA}{Total\ BA} \quad \textbf{(Eq. 9)}$$

$$Forest\ BA_{t,c} = Total\ BA_{t,c} * Forest\ BA\ Proportion \quad \textbf{(Eq. 10)}$$

$$NonFor\ BA_{t,c} = Total\ BA_{t,c} - Forest\ BA_{t,c} \quad \textbf{(Eq. 11)}$$

Where *t* corresponds to the simulated time (year) and *c* to the grid cell.

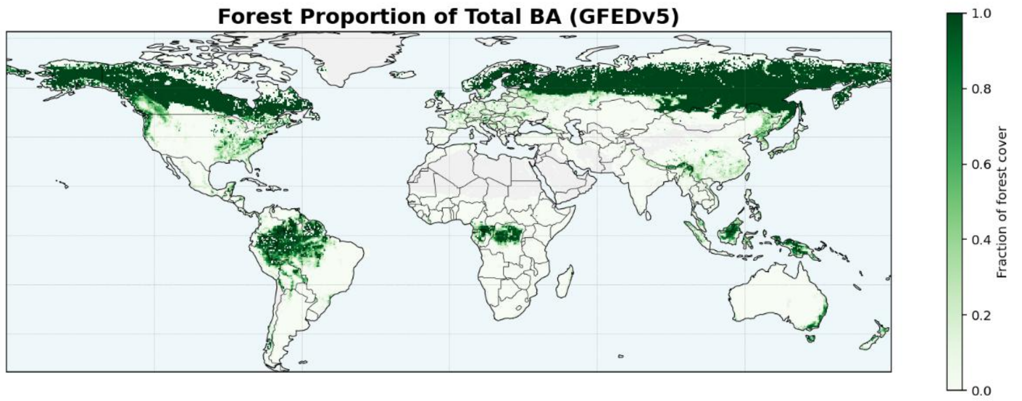

Figure S2. Forest Proportion of Total BA used in the proportion approach based on GFEDv5.1.

1.2.6. Carbon emissions

Carbon emissions are derived from burned areas and dynamic fuel consumption which is itself derived from combustion completeness and fuel load.

Fuel load is extracted from the global fuel characteristic dataset (McNorton & Di Giuseppe, 2024) which includes foliage and wood fuel load, including both alive and dead components. Fuel load has been increasing with a trend of +4.5 Pg.yr-1 from 2010-2019. Splitting the fuel load into fuel types is required since each component of vegetation has different CC factors.

For forests, we sum the fuel consumption values of all the four fuel types while for non-forests we sum stem and leaf values. Additionally, we add the fuel load of soil carbon stock in the boreal region. While soil carbon stock is the main fuel types of boreal fires it is not provided in the fuel load dataset. We follow the approach of Park et al. 2023 which add in the boreal regions an average value of 2200 $gC.m^{-2}$ (Park et al., 2023).

CC values which were extracted from van Wees et al., (2022). Rather than assigning fixed CC values per biome, this approach scales CC dynamically using mean annual VPD, which reflects fuel dryness and atmospheric demand for moisture. CC was scaled linearly by VPD values to account for the impact of dryness/wetness of the fuel. While GFED5 and related fire models use soil moisture content to scale CC, the variable cannot be consistently emulated by STITCHES and hence we consider VPD as an appropriate substitute or proxy of fuel flammability.

Table S4. Combustion completeness factors per biome and fuel types

**Biome**	**Stem**		**Leaf**		**Coarse**		**Litter**	
	Min	Max	Min	Max	Min	Max	Min	Max
**Boreal**	10	30	90	100	30	70	70	100
**Temperate**	30	50	90	100	20	60	70	100
**Tropical**	20	40	90	100	30	70	80	100

We derive from the GFEDv5 data the fuel consumption per grid cell for forest fires and non-forest fires for the last available year (2019) (Eq. 12). The dynamic FC estimated with our methodology and the historic FC from GFEDv5 have significant differences (Figure S4 vs. Figure S3). Hence to ensure consistency between BA and CE products, we use the GFEDv5 FC as basemap and project onto it the change ratio per grid cell in the simulated period from our estimate (Eq. 13-14). This effectively scales dynamically given changes in VPD the GFED-derived FC.

$$FC\_GFED_{LT,C,Y,HIST} = \frac{CE_{LT,C,Y,HIST}}{BA_{LT,C,Y,HIST}} \quad \textbf{(Eq. 12)}$$

$$FC\_Ratio_{LT,C,Y,SIM} = \frac{FC_{LT,C,Y,SIM}}{FC_{LY,C,2020,SIM}} \quad \textbf{(Eq. 13)}$$

$$FC\_Scaled_{LT,C,Y,SIM} = FC\_GFED_{LT,C,2019} * FC\_Ratio_{LT,C,Y,SIM} \quad \textbf{(Eq. 14)}$$

Where *FC* is the fuel consumption ($gC.m^{-2}$), *CE* is the carbon emissions (gC), *BA* is the burned area (m^{-2}), *LT* is the land type (forests or non-forests), *C* is the grid cell, *Y* is the year (2019), *HIST* is the historic period (2002-2019) and *SIM* is the projected period (2020-2100).

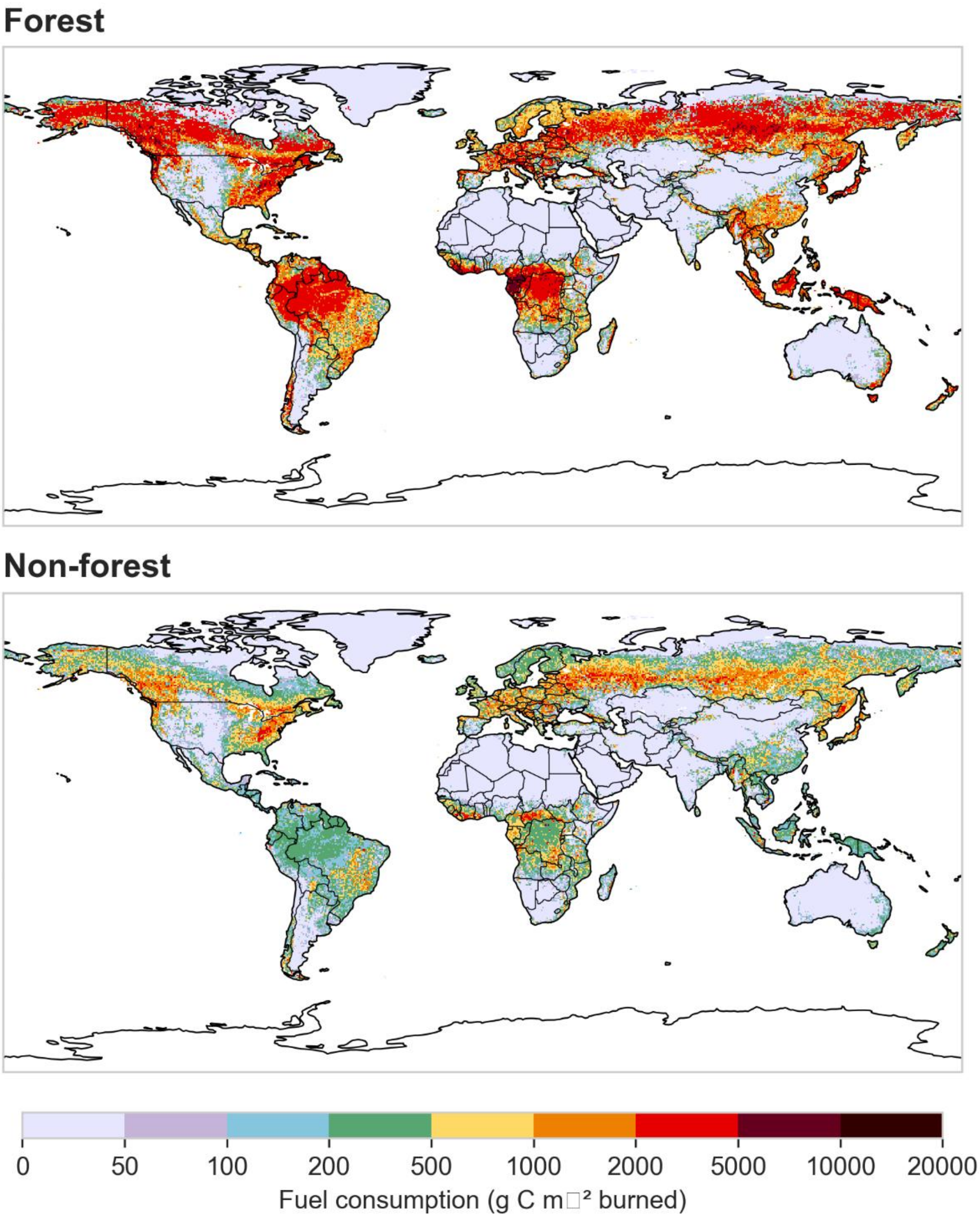

Figure S3. Fuel consumption (gC.m2 burned) derived from McNorton et al. (2024) fuel load datasets and CC factors from van Wees et a. (2022) and used as derive dynamic changes FC in simulated period considering VPD scaling on CC

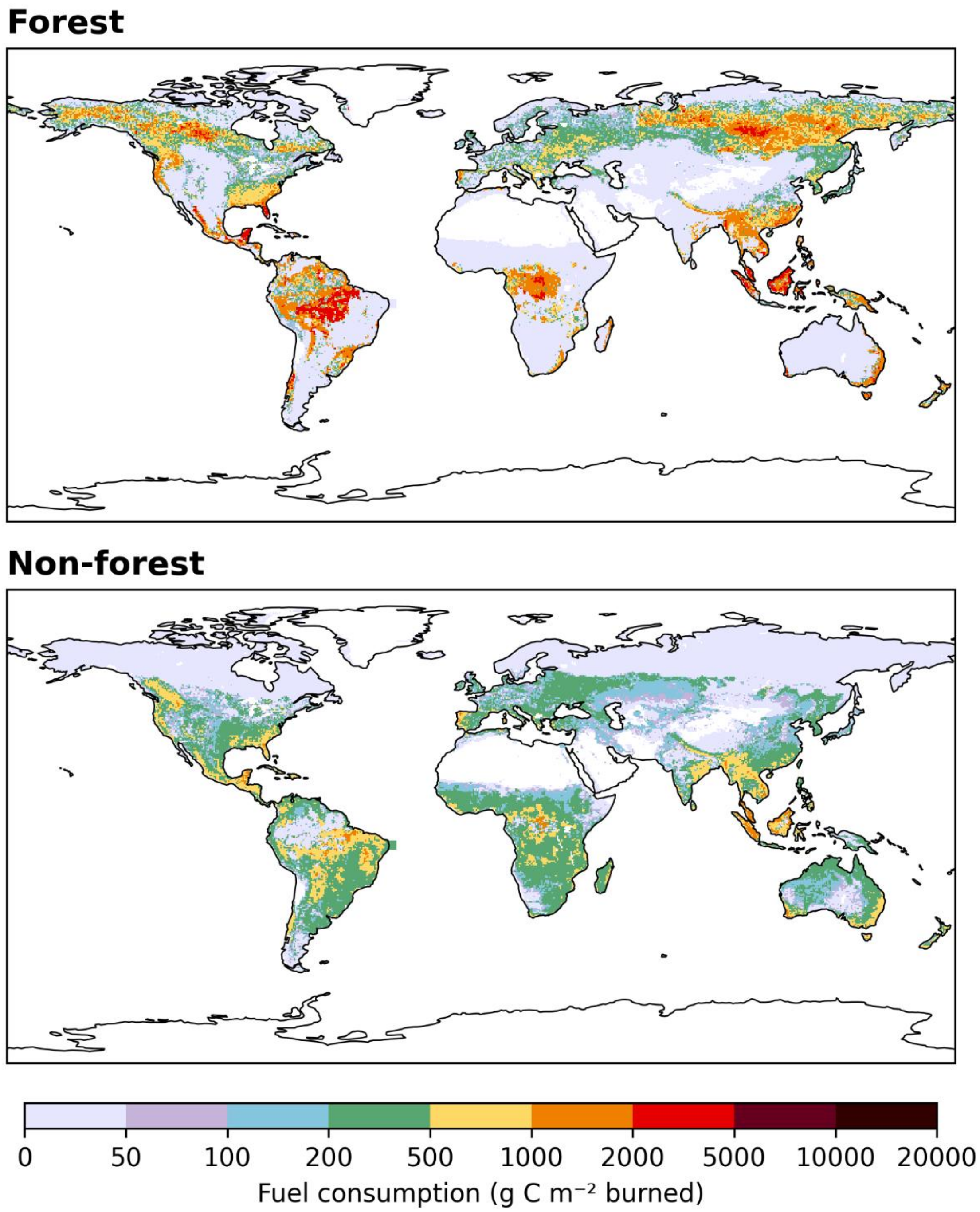

Figure S4. Fuel consumption (gC.m2 burned) derived from GFEDv5 and used as basemap to project dynamic changes to FC in simulated period.

1.3. Scenario design

We extract from the simple climate model Hector the trajectories of temperature and CO_2 concentration for the four scenarios of the scenario design (Figure S5). The scenario "SSP1-2.6" is marked by a low overshoot over the 1.5°C goal, reaching a maximum of 1.57°C in 2065 before returning to 1.48°C in 2100.

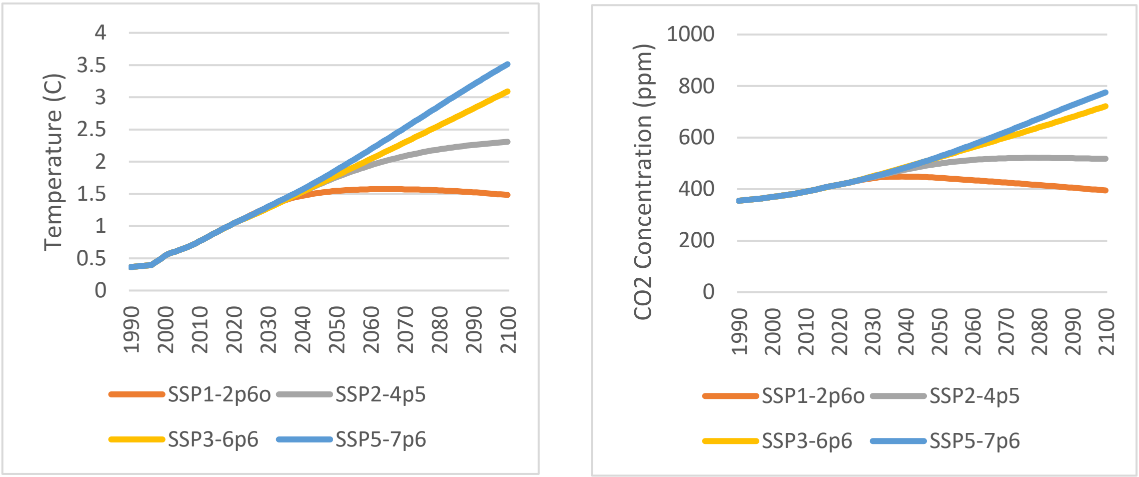

Figure S5. Temperature trajectory (left) and CO2 concentration (right) of the SSP-RCP scenarios modelled by GCAM & Hector

1.4. Factorial decomposition

We conduct a factorial decomposition following the approach of (Wu et al., 2021) which differentiated between drivers and limiting factors of BA. In the analysis of drivers, we conduct runs where all but one group of variables is fixed while in the analysis of limiting factors we conduct runs where all but one groups of variables vary. We group the predictors in four groups and rerun the BA prediction as shown in Table S5.

Table S5. Factorial decomposition runs for limiting factors and drivers. A prefix "+" means the group of variables is varying/dynamic while a prefix "-" means it is fixed at the 2002-value for the historic period and at the 2020-value for future period

**Variable group**	**Default run**	**Limiting factors**				**Drivers**			
		L1	L2	L3	L4	D1	D2	D3	D4
Climate	+	-	+	+	+	+	-	-	-
Land use	+	+	-	+	+	-	+	-	-
Vegetation	+	+	+	-	+	-	-	+	-
Socio-economic	+	+	+	+	-	-	-	-	+

2. Supplementary Material: Results

2.1. Historical burned area prediction (GLMs)

The final GLM was selected after a process of filtering out predictor variables which didn't improve the GLM performance and/or were correlated to others variables. Here we report additional results, information on the final GLM model, complementary details on the other relevant GLMs and results of out-of-sample tests and a GPP sensitivity analysis.

2.1.1. Final GLM additional results

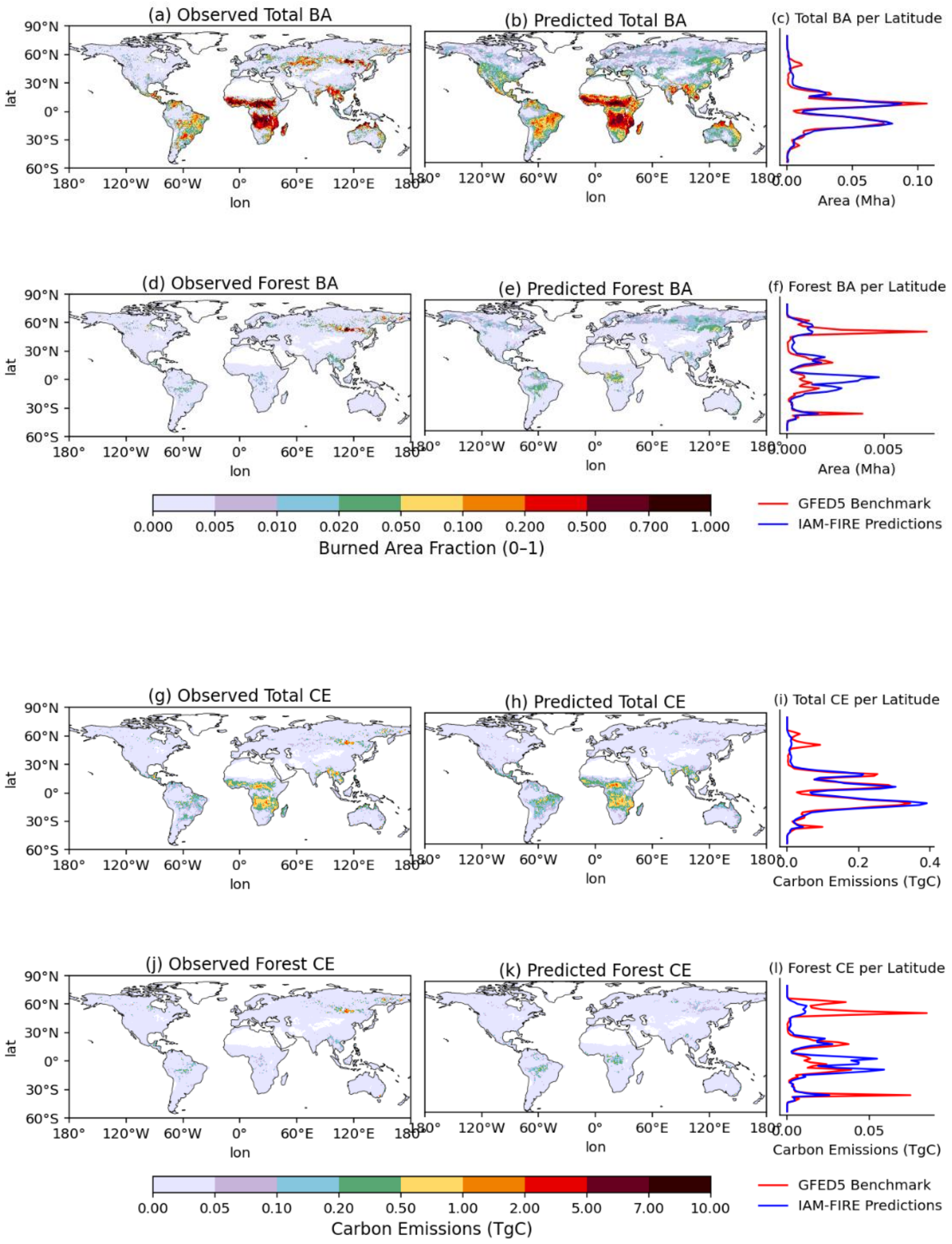

Figure S6. Annual values for 2003 of observed and observed response variables and mean latitudinal distributions over historic period (2002-2019) for total BA (a-c), forest BA (d-f), total CE (g-i), and forest CE (j-l). Maps for BA are shown in fraction (0-1) and latitudinal bands in Mha. Carbon emissions are reported in TgC.

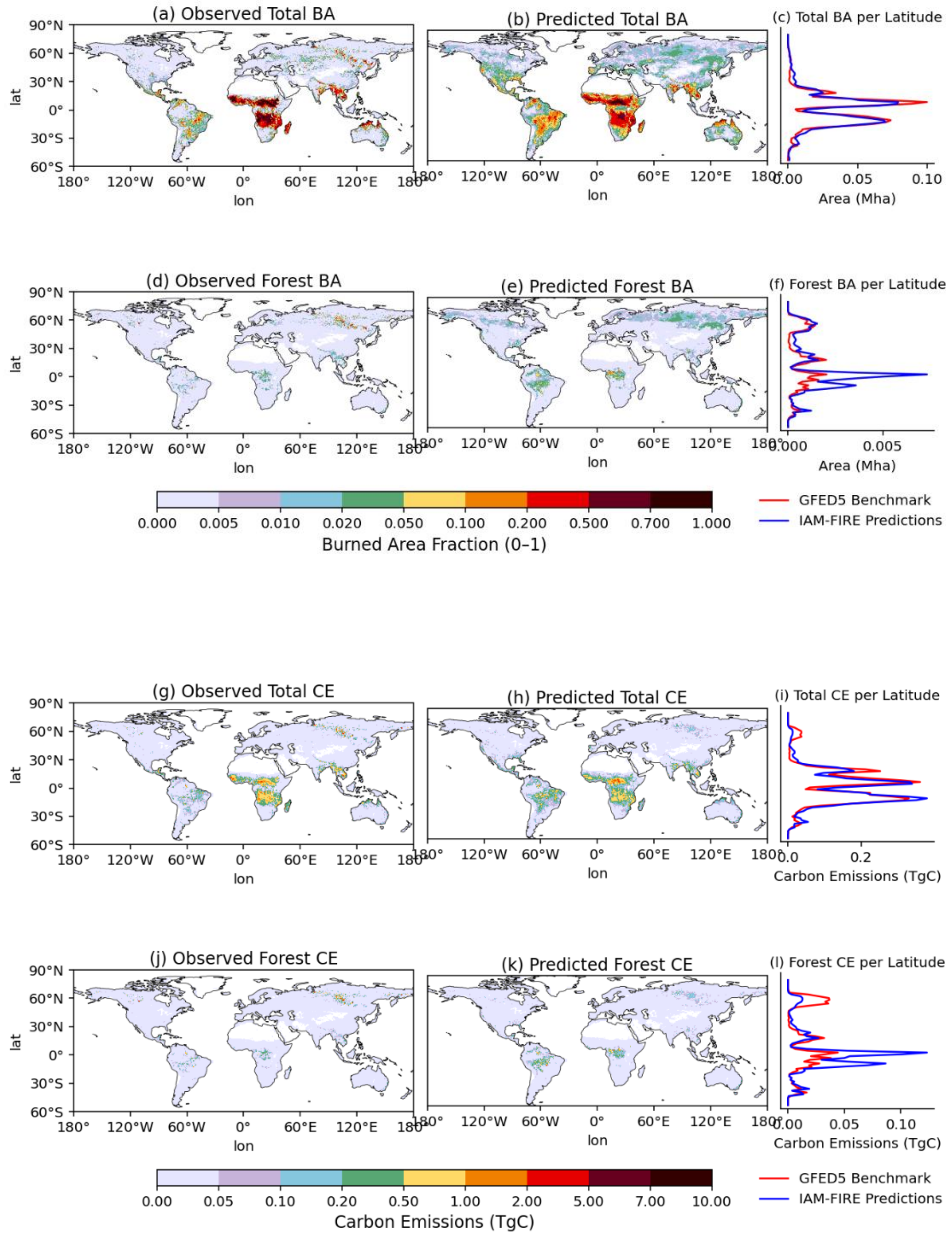

Figure S7. Annual values for 2016 of observed and observed response variables and mean latitudinal distributions over historic period (2002-2019) for total BA (a-c), forest BA (d-f), total CE (g-i), and forest CE (j-l). Maps for BA are shown in fraction (0-1) and latitudinal bands in Mha. Carbon emissions are reported in TgC.

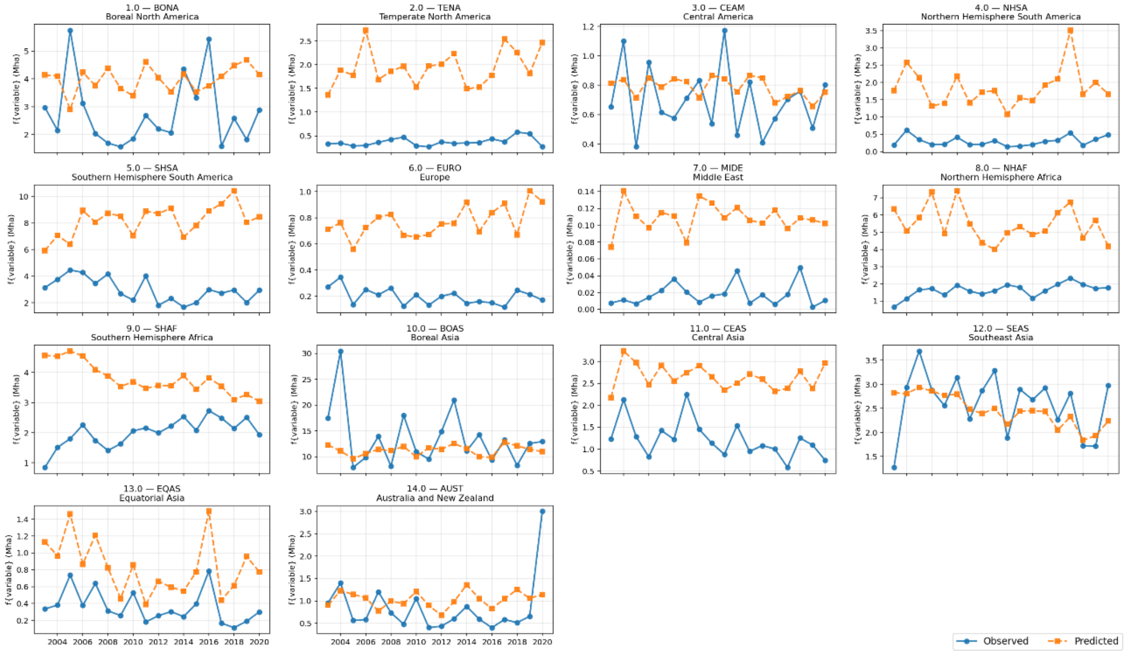

Figure S8. Regional trends for total burned areas for observations and predictions from 2002-2019

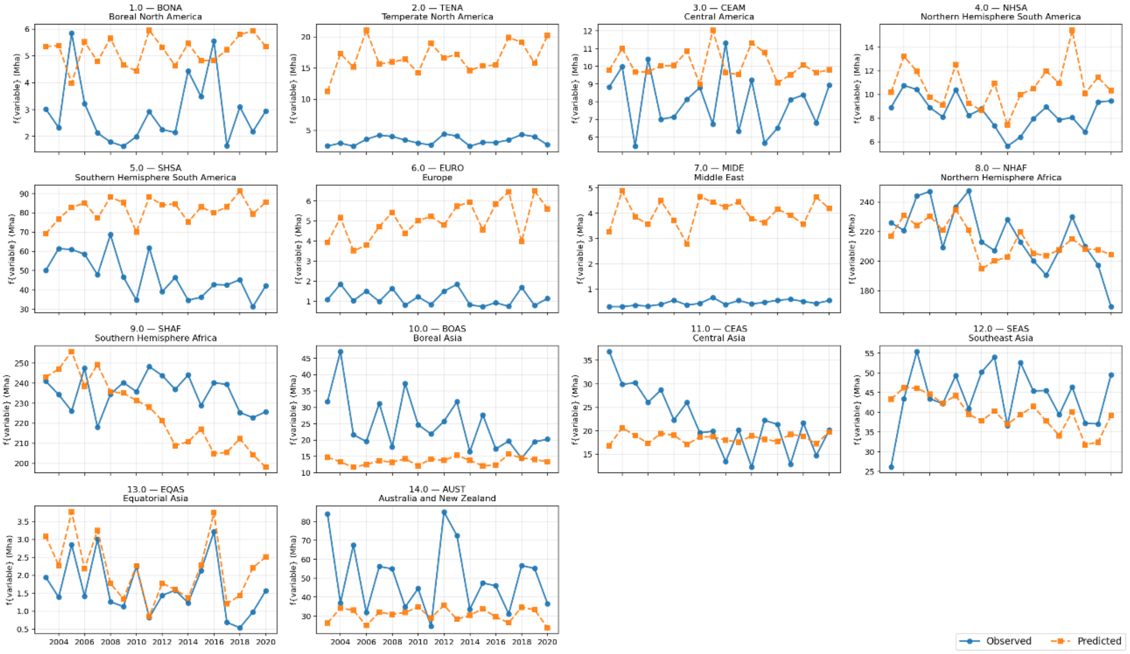

Figure S9. Regional trends for forest burned areas for observations and predictions from 2002-2019

2.1.2. Final GLM performance

For the initial set of predictors compiled over the historic period, we tested for non-linear relationship among the predictors using a Generative Additive Models (GAMs) which demonstrated high explanatory power, accounting for 80.8% of the total deviance (Pseudo R2 = 0.81). The model helped identifying four variables that are both significant and exhibit high levels of non-linearity (according to the EDoF parameter): VPD, NDD seasonality, grassland and HDI (Table S6).

Table S6. Summary statistics of the GAMS used to identify non-linear relationships between predictors and BA

FEATURE NAME	EDOF	P-VALUE	SIGNIFICANCE
**VPD_LOG**	**10.4**	0.00e+00	*** (High)
**NDD_LOG**	7.1	2.42e-08	*** (High)
**NDD_SEASON_LOG**	**11.3**	2.08e-07	*** (High)
**PR_SUM_MONTH_LOG**	4.7	1.52e-05	*** (High)
**GPP_LOG**	6.5	2.33e-13	*** (High)
**TPI**	7.4	872	Not Significant
**VRM**	5.4	3.41e-05	*** (High)
**GRASSLAND**	**13.4**	0.00e+00	*** (High)
**SHRUBLAND**	9.7	643	Not Significant
**CROPLAND**	12.5	999	Not Significant
**HDI**	**12.6**	0.00e+00	*** (High)
**GRAZING_PRESSURE**	8.8	0.00e+00	*** (High)

We implemented polynomial terms to these four variables in the GLM. From the initial set of predictors, the forest variable was removed due to a high correlation with GPP. The GPP seasonality index, wind speed, TPI and shrubland were removed because they did not improve the model's performance. The VPD polynomial term was not added as it was not statistically significant in the GLM. Final VIF values (VIF_{max} = 4.4) demonstrate a low level of multicollinearity among predictors (Table S7).

Positive linear relationships (z-score) are observed for VPD (28.3), NDD (18.2), precipitation (15.6), and GPP (30.6), while negative linear relationships are observed for VRM (−16.7) and grazing pressure (−26.0). Notably, the model captures significant non-linear relationships for several key drivers (Figure S10). Grassland area emerges as the strongest overall predictor (z=34.5), but its negative quadratic term (−25.3) indicates a "hump-shaped" relationship where fire risk eventually saturates or declines at extremely high grassland fractions. Similarly, HDI shows a strong negative linear pressure (−15.3), but its positive quadratic term (11.2) suggests a U-shaped recovery or a flattening rate of fire suppression at the highest levels of development.

NDD-seasonality also exhibits non-linear behaviour; while its linear effect is weakly negative (−5.7), its strong negative quadratic term (−19.3) implies that fire risk is severely constrained at both ends of the seasonality spectrum. Overall, high levels of human development and grazing activity continue to play a crucial role in counteracting the increasing risks posed by climate change. These risks materialize directly through drier conditions (reflected by VPD and NDD) and indirectly through the accumulation of fuel via CO2 fertilization and increased vegetation productivity (reflected by the strong positive influence of GPP). The positive precipitation coefficient illustrates the role of fuel-limitation in dry or grass-dominated landscapes where increased moisture stimulates vegetation growth and fuel load, despite the known dampening effect of rainfall on fuel flammability at higher levels (Li et al., 2024).

Table S7: Summary statistics of the final GLM model used to predict burned area. Predictors included were all significant at Pr (>|t|) < 1.97e-11. VPD: Vapor Pressure Deficit, NDD: Number of Dry Days, GPP: Gross Primary Productivity, VRM: Vector ruggedness measure, HDI: Human Development Index

GROUP	VARIABLE	COEFFICIENT	STD. ERROR	Z	P>|Z|	[0.025]	[0.975]	VIF
	Intercept	-27.7809	0.857	-32.406	0	-29.461	-26.101	2237.28
**CLIMATIC FACTORS**	VPD (log)	0.5692	0.02	28.308	0	0.53	0.609	2.05
	NDD (log)	3.1532	0.173	18.177	0	2.813	3.493	2.03
	NDD Seasonality (log)	-0.1889	0.033	-5.721	0	-0.254	-0.124	2.77
	NDD Seasonality^2	-0.5113	0.026	-19.3	0	-0.563	-0.459	

	Precipitation (30-Day sum)	0.6128	0.039	15.564	0	0.536	0.69	4.45
**VEGETATION**	GPP (log)	0.86	0.028	30.63	0	0.805	0.915	2.53
**TOPOGRAPHY**	VRM	-128.711	7.69	-16.738	0	-143.783	-113.639	1.16
**SOCIOECONOMIC**	HDI	-8.6622	0.567	-15.28	0	-9.773	-7.551	1.47
	HDI^2	4.9512	0.443	11.186	0	4.084	5.819	
**LAND USE**	Grassland	7.2221	0.209	34.524	0	6.812	7.632	1.61
	Grassland^2	-5.2381	0.207	-25.285	0	-5.644	-4.832	
	Cropland	0.3985	0.08	4.972	0	0.241	0.556	2.14
	Grazing Pressure	-5.6444	0.217	-26.007	0	-6.07	-5.219	1.88

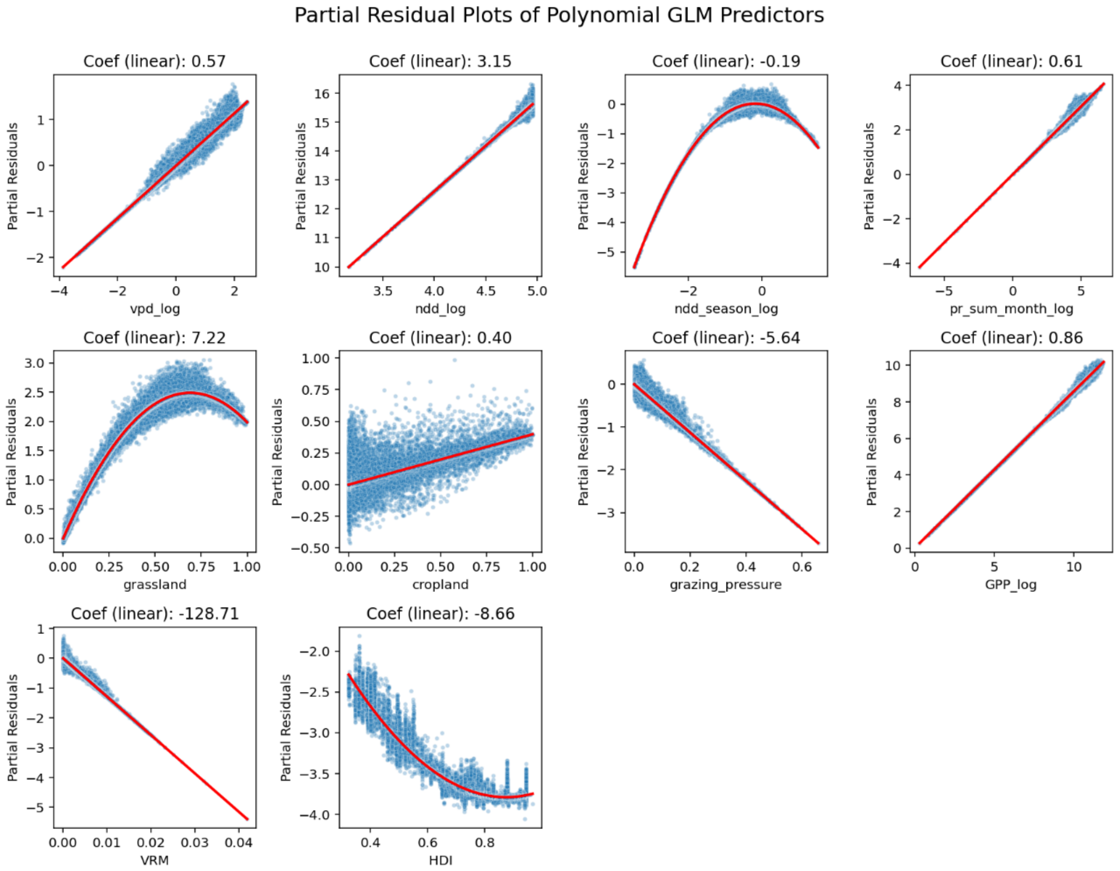

Figure S10. Partial residual plots of the predictor variables of the final GLM. Colours correspond to the category (vegetation, topography, land uses, climate and socio-economics).

2.1.3. Out-of-sample validity checks

The rolling window hindcast results demonstrate that the fire prediction model is highly robust to out-of-sample tests throughout the historic period (2002–2019) (Table S8). The predictive performance remains stable across all 16 validation windows, as shown by the tight clustering of the Pseudo-R2 values (Mean = 0.668, σ=0.0046). This consistency, paired with stable error metrics (RMSE and NME), indicates that the model is not prone to overfitting and effectively captures the underlying physical drivers of fire activity.

Because the model maintains high predictive skill when applied to different temporal subsets, it can be considered a reliable tool for long-term trend analysis and future projections, as the identified relationships between climate, vegetation, and human activity appear temporally

invariant. Specifically testing the 2017–2019 window (the most recent period available in our driver dataset) yielded an R2 of 0.668 and an RMSE of 0.037, confirming that the model effectively captures fire trends in "unseen" modern climate conditions.

Table S8. Summary statistics of the rolling hindcast results for out-of-sample validity checks

METRIC	MEAN	STD DEV	MIN	MAX
**PSEUDO-R2**	0.668	0.0046	0.664	0.682
**RMSE**	0.775	0.0036	0.737	0.876
**NME**	0.478	0.0134	0.460	0.508

2.1.4. Modified GLMs

We present the results of variations of the final GLM with and without additional predictors (Table S9). First, we remove HDI since while it has a strong suppressing effect on BA, such a composite indicator does not capture the specific processes underlying the suppressing effects. Removing the HDI caused a significant drop in performance (Pseudo R2 fell from 0.749 to 0.726), confirming that socioeconomic factors are critical for explaining global burned area patterns.

Second, we add population density as a predictor to test the extent to which the influence of human factors on BA depends on density and/or how it dampens the suppressing effect of HDI. We also add a proxy of fragmentation which has been shown to interact with BA in divergent directions which was computed as the number of land uses in a grid cell above the 10% threshold. The inclusions of fragmentation and population density – both separately and together – do not significantly improve the model beyond a slightly superior performance with Pseudo R2 of 0.75 (against 0.749 in the final GLM).

Last, we report results from combination of predictors and GLMs with an interaction term between HDI and population density to explore the possible relation where human development suppressing effects are mediated by population density. The modified GLM does not present meaningful performance improvements compared to the final one either.

Table S9: Summary statistics of the modified GLMs

GLM VERSION	MODIFICATION	PSEUDO R2	RMSE	NME
**FINAL GLM**	Final (Polynomial)	0,749	0,064	0,434
**NO HDI**	Final – HDI	0,726	0,068	0,463
**FRAGMENTATION**	Final + Frag_index	0,749	0,064	0,434
**POPULATION**	Final + Pop_density	0,750	0,064	0,433
**FRAGMENTATION + POPULATION**	Final + Frag + Pop	0,750	0,063	0,433
**POPULATION × HDI**	Final + HDI * Pop_density	0,750	0,064	0,433

2.1.5. GPP sensitivity analysis

The GPP estimates used for future projections assume a constant fAPAR for future periods. To assess the relevance of this assumption, we run a sensitivity analysis of GPP for the historical period with alternative fAPAR estimates. The GIMMS FPAR4g (Zhao et al., 2024) provides data from 1982 up to 2022. The final GLM uses GPP projections based on fAPAR for the default latest years of the historical period (2018-2019).

For the sensitivity analysis, we first run the P-Model for the historical period using fAPAR from past periods (1983-2001) to assess the impact on GPP. Then we run the GLM with the modified GPP to assess the final impact on burned area predictions of the past fAPAR estimates. Figure

S11 shows the spatial difference introduced in GPP by using past fAPAR compared the default. GPP is slightly higher in regions which experienced greening between the 1990s and the 2010s such as Eastern China and Europe and lower in regions that experienced forest loss and degradation such as South America. Still the differences are constrained between -8 and 8 gC.m^2.year.

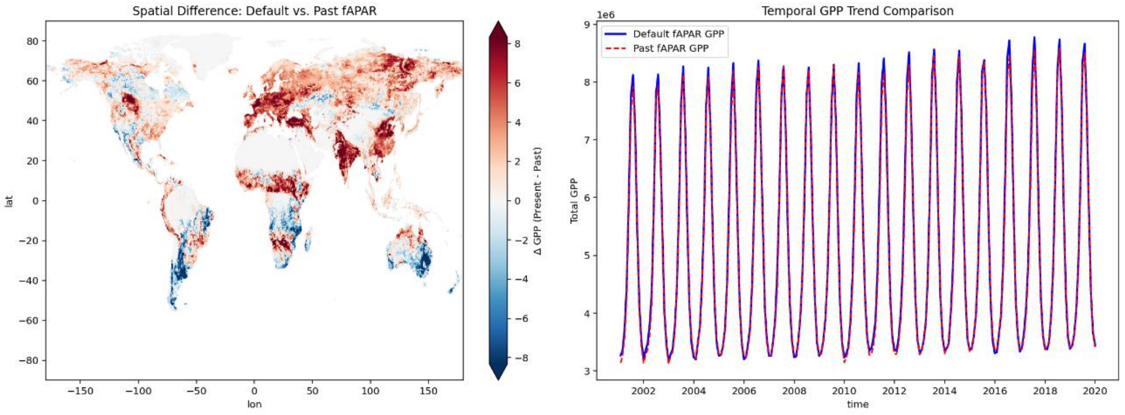

Figure S11. Sensitivity analysis of GPP estimates using default fAPAR from default historical period (2018-2019) vs. past period (1983-2001). Left panel represents average spatial difference and right panel represent the temporal trend of the global sum of GPP estimates.

Table S10 and Figure S12 illustrate the result of the modified GLM with the GPP based on past fAPAR. While the t-value coefficient of GPP remains similar in both GLM as well as the overall statistical performance of the GLM, the sensitivity analysis shows how the temporal dynamic of BA is modified by the alternative assumption of fAPAR, driven by the differences in GPP illustrated in Figure S11.

Table S10. Results of GLM sensitivity analysis using GPP estimates based on past fAPAR (1983-2001)

Variable	GPP t-value (GLM coefficient)	Pseudo R2	RMSE	NME
Default GPP	30.6	0.749	0.0637	0.434
Modified GPP	28.7	0.748	0.0639	0.435

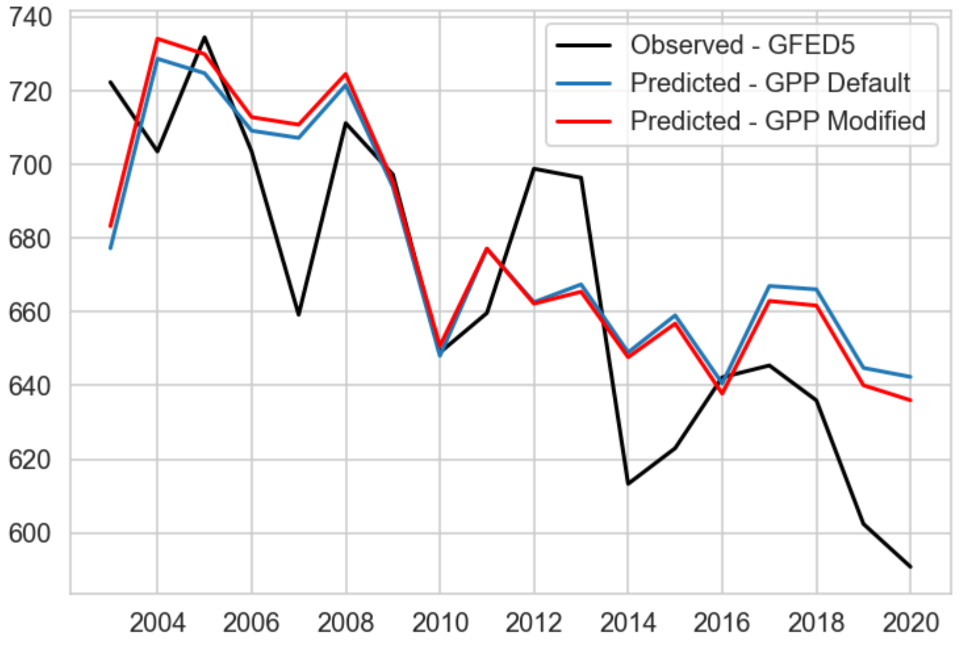

Figure S12. Global BA trends for historic period (2002-2019) for observation data (GFED5), final GLM with default GPP, and modified GLM with GPP based on past fAPAR

2.2. Future burned area per region

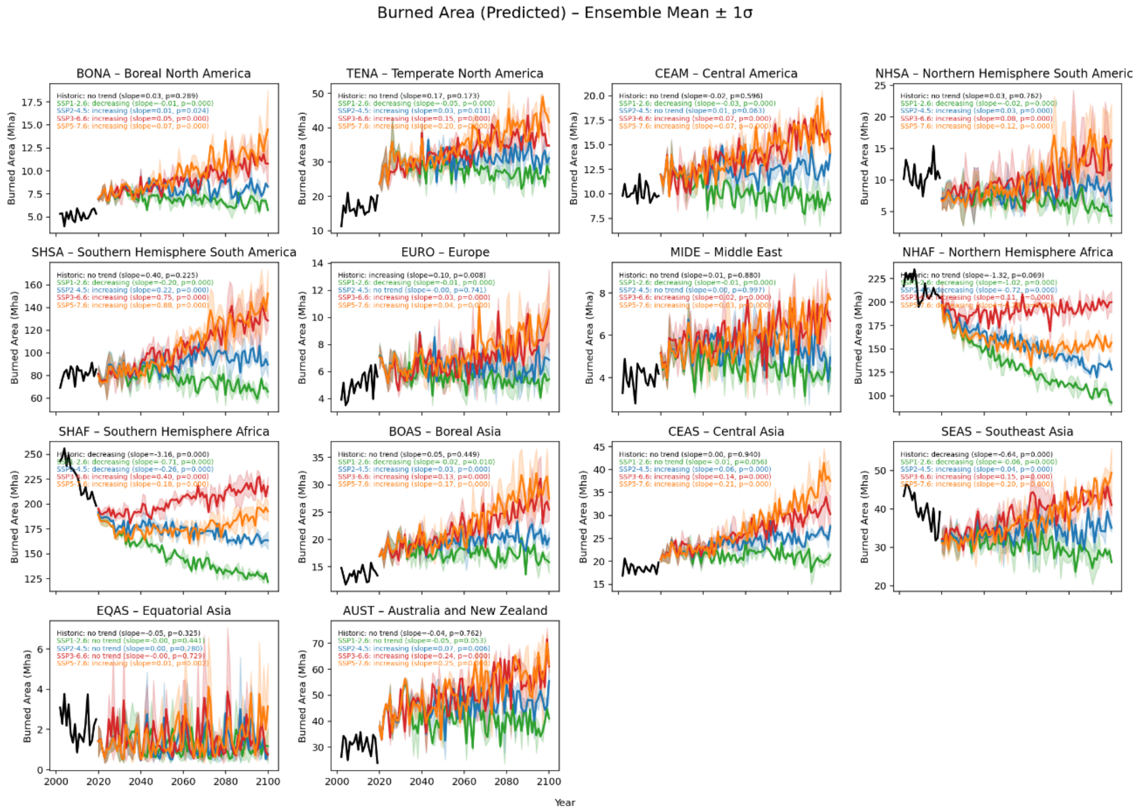

Figure S13: Total burned area per GFED regions

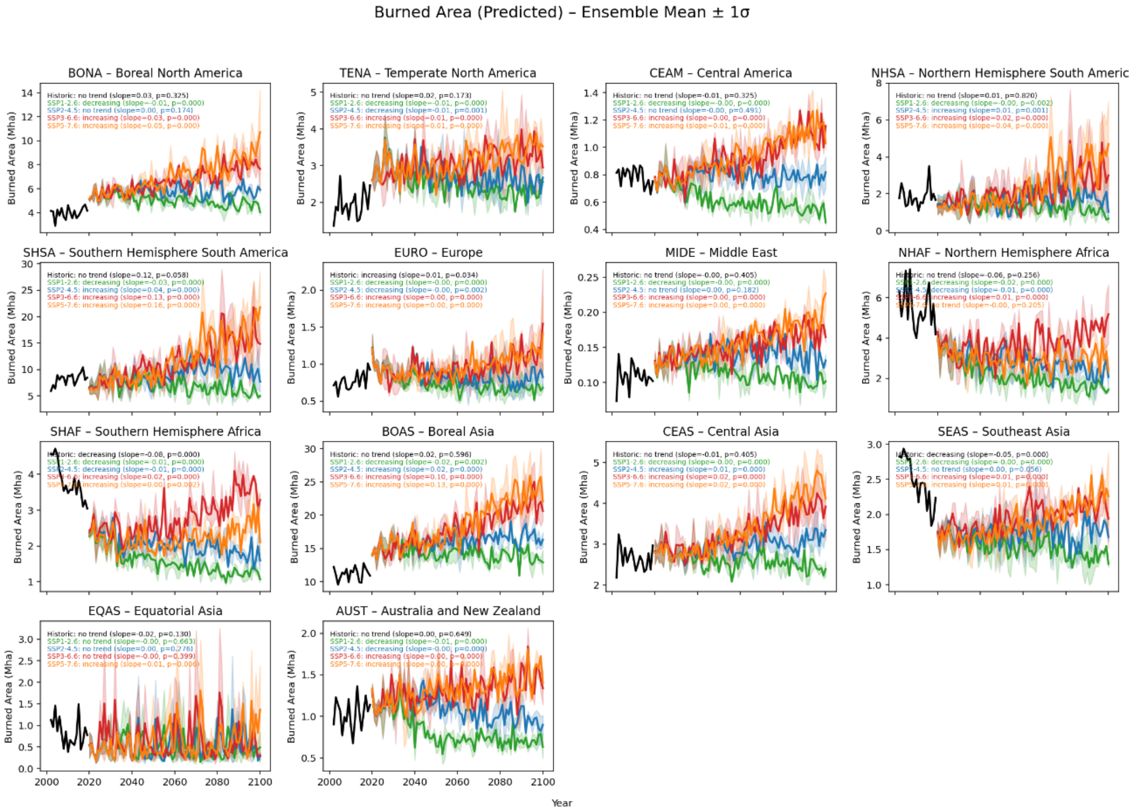

Figure S14. Forest burned area per GFED regions

2.3. Future carbon emissions per region

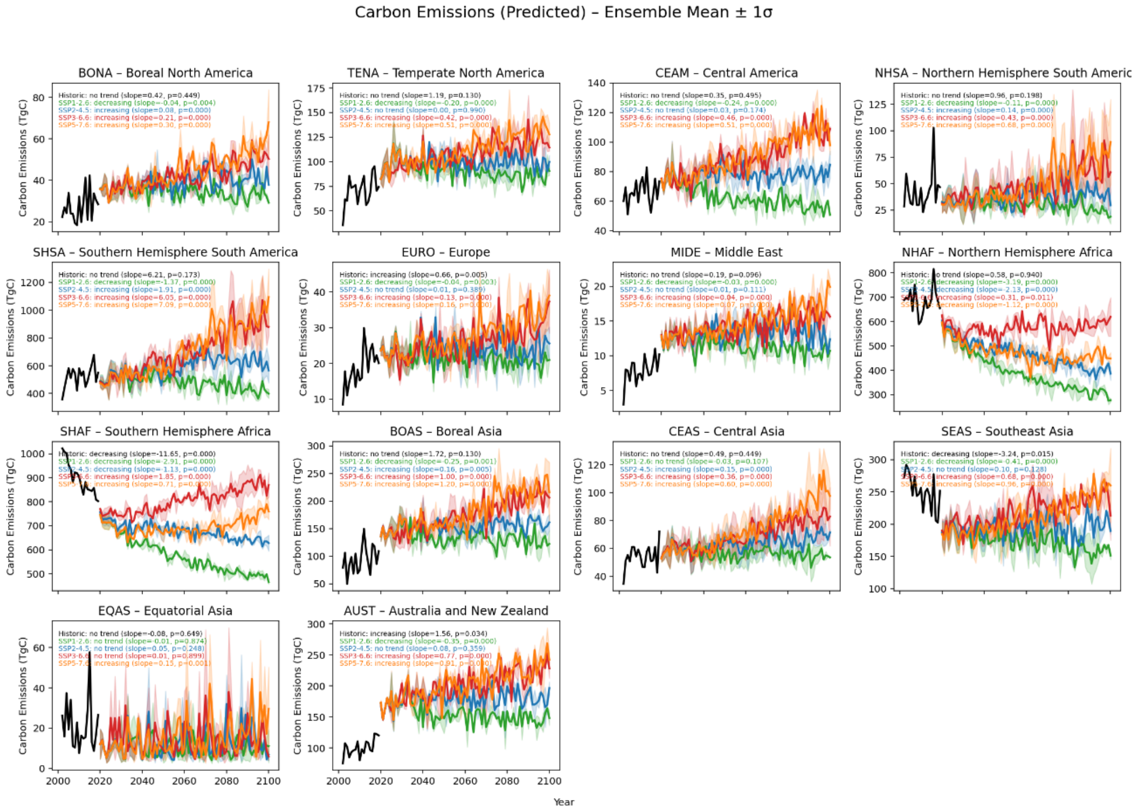

Figure S15. Total carbon emissions per GFED regions

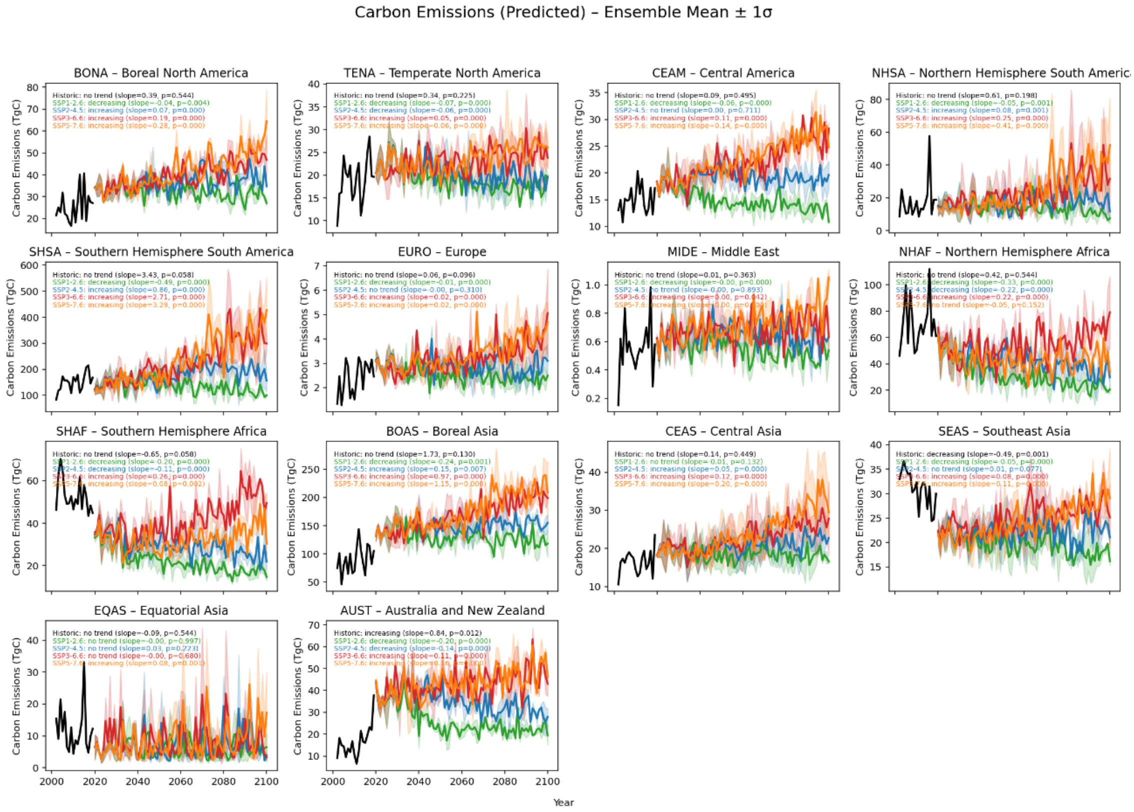

Figure S16. Forest carbon emissions per GFED regions

2.4. Trends in drivers of fire impacts

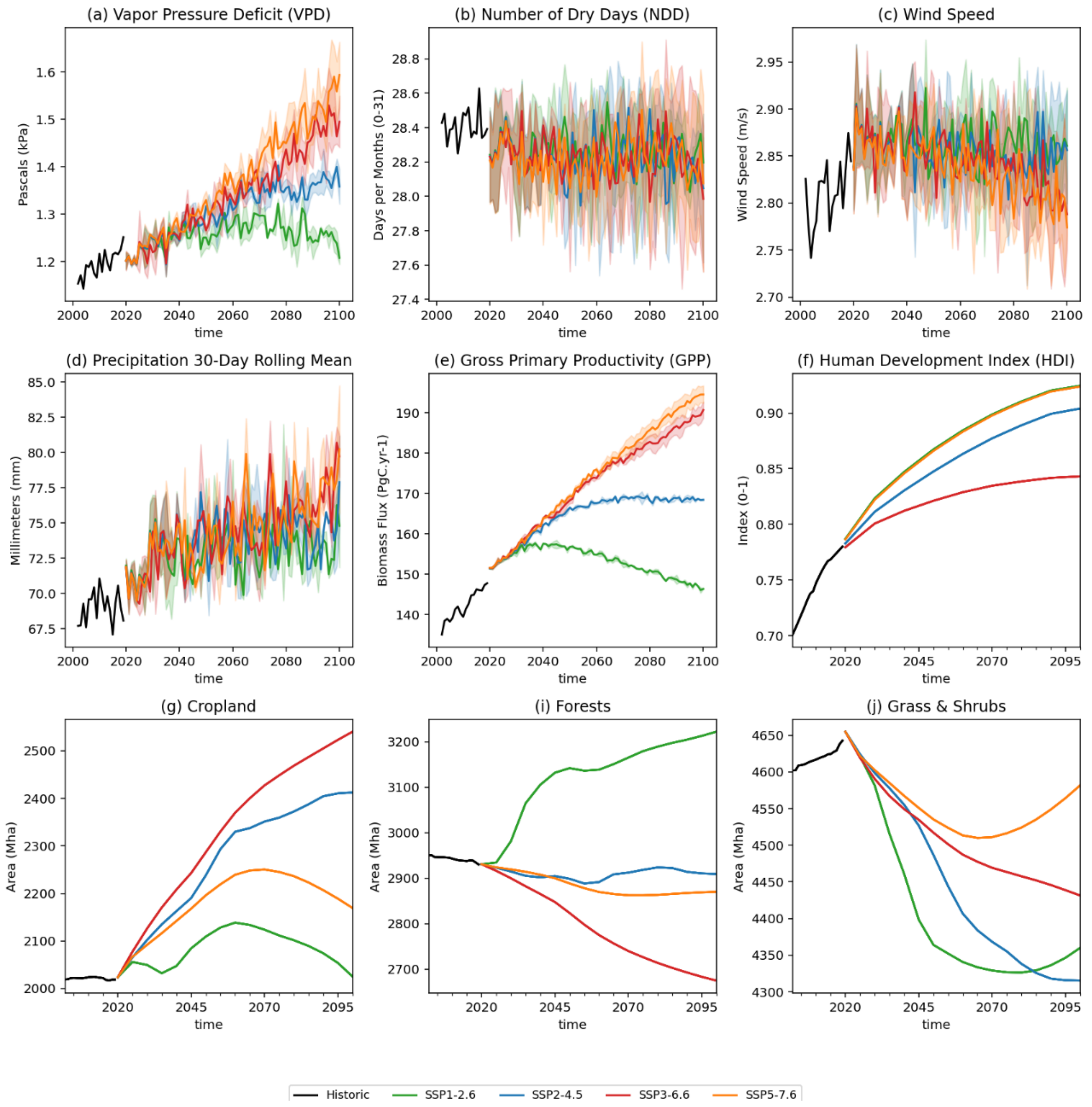

Figure S17. Trends of the drivers of total burned area

2.5. Factorial decomposition

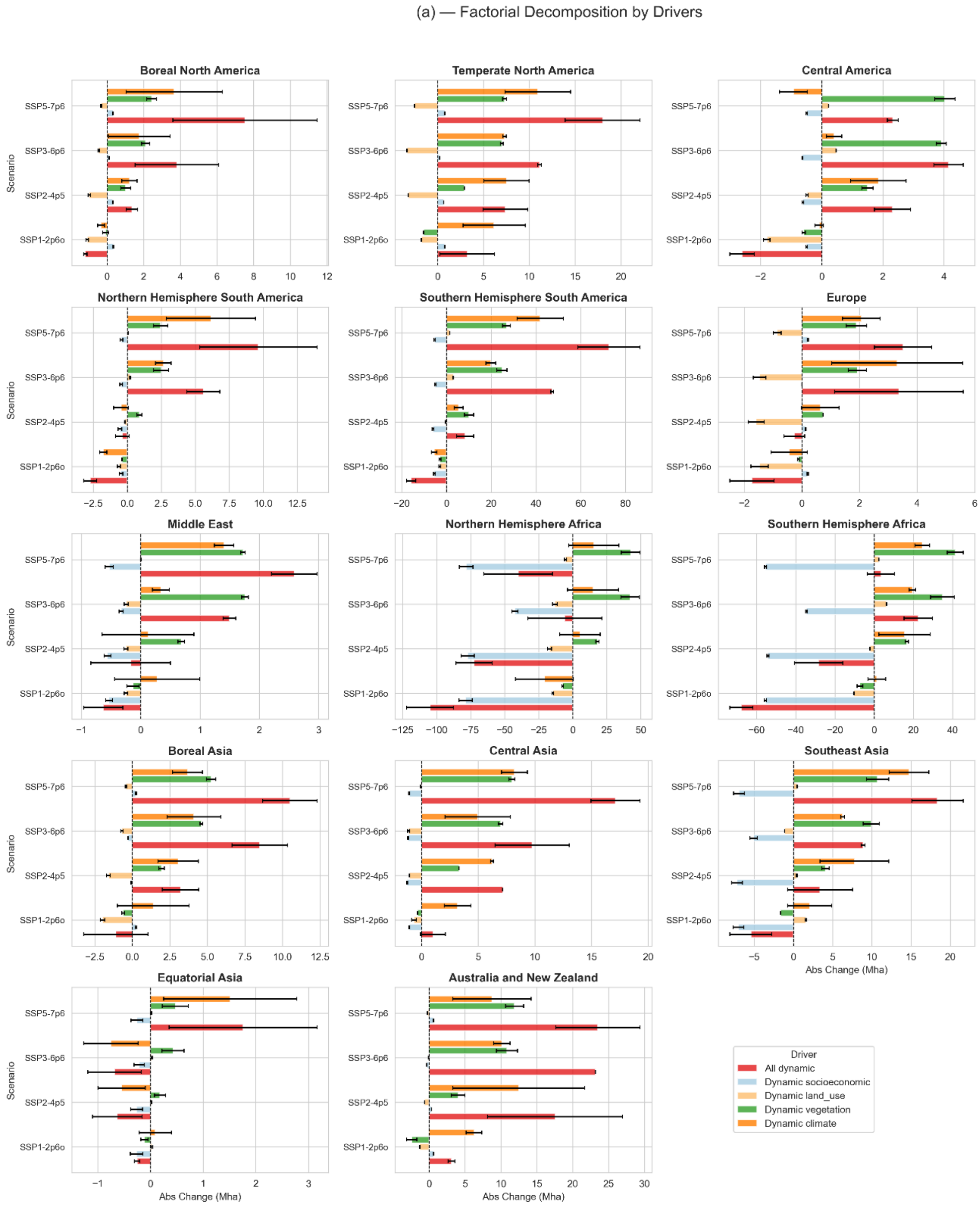

Figure S18. Factorial analysis of drivers per GFED regions. Error bars represent the range between the two ESMs.

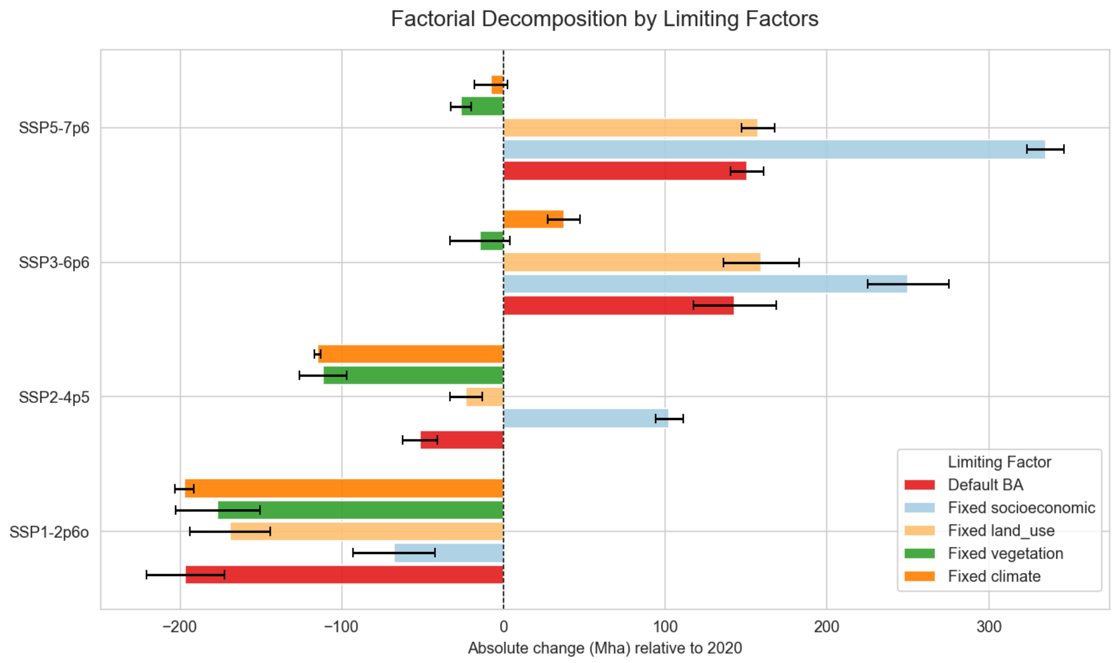

Figure S19. Factorial decomposition of limiting drivers for global scenarios. Error bars represent the range between the two ESMs.

(a) — Regional Factorial Decomposition (Limiting Factors)

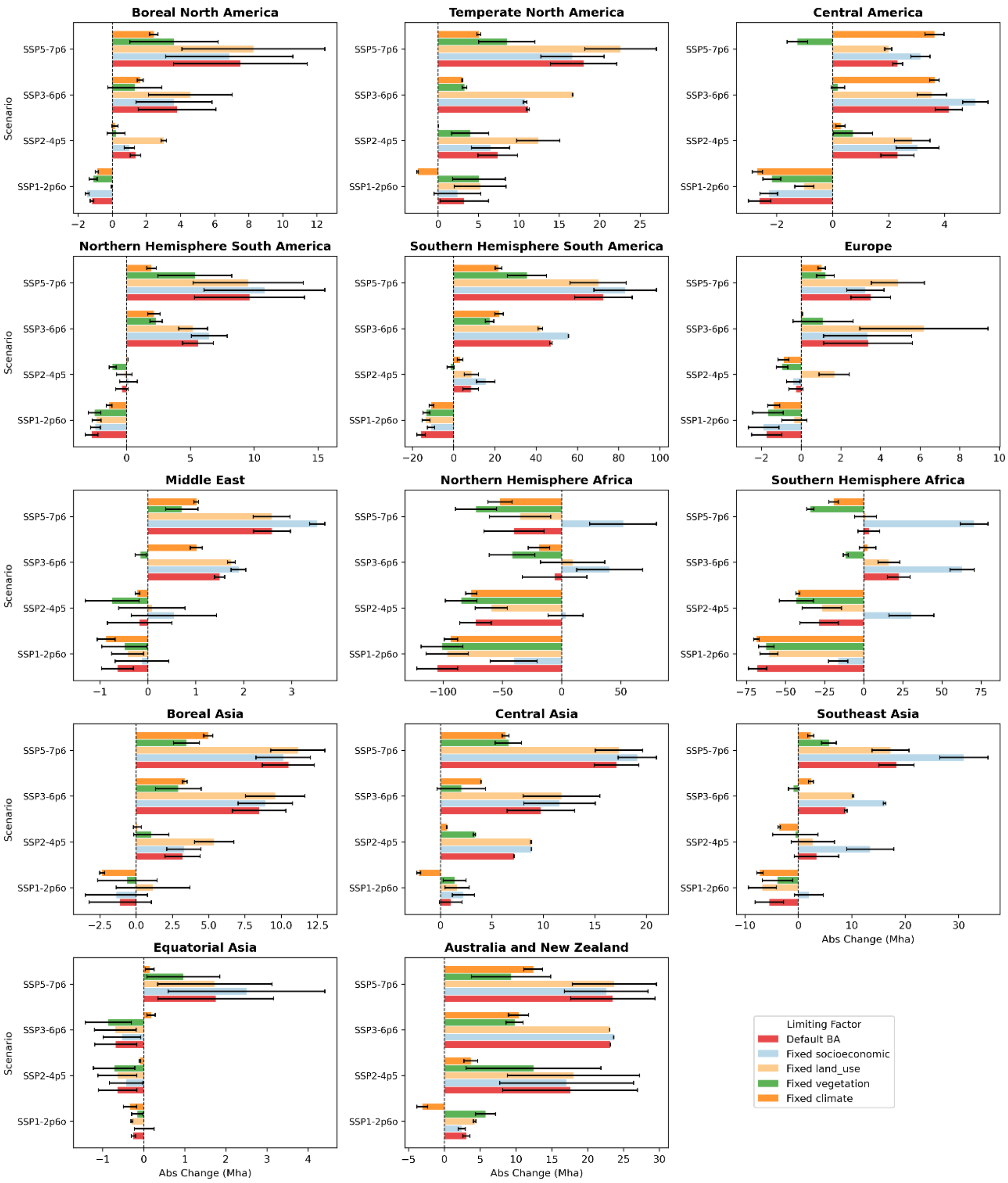

Figure S20. Factorial analysis of limiting factors per GFED regions. Error bars represent the range between the two ESMs.
